# An international Delphi consensus for reporting of setting in psychedelic clinical trials

**DOI:** 10.1038/s41591-025-03685-9

**Published:** 2025-06-03

**Authors:** Chloé Pronovost-Morgan, Kyle T. Greenway, Leor Roseman, The ReSPCT Experts, Jacob S. Aday, Gabrielle Agin-Liebes, Kirran Ahmad, Helena Aicher, Marc Aixalà, Simon Amar, Draulio B. de Araujo, Frederick S. Barrett, Alexander Belser, Marc Blainey, Michael Bourne, Ronald Bowman, Joost Breeksema, Kayla Breelove Carter, Jacqueline Bussey, Cesar Camara, Nicholas Canby, Jimena Chalchi, James Close, Tonie Cox, Pedram Daraeizadeh, Alan K. Davis, Sara de la Salle, Neşe Devenot, Erika Dyck, David Erritzoe, Houman Farzin, Phoebe Friesen, Nicolas Garel, Josephine Giblin, Tasha Golden, George Greer, Natalie Gukasyan, Jeffrey Guss, Emma Hapke, Ido Hartogsohn, Friederike Holze, Hannes Kettner, Adam Knowles, Nicolas Langlitz, Alexandre Lehmann, Michael Lifshitz, Philippe Lucas, Katherine MacLean, Orla Mallon, Olivia Marcus, Jerónimo Mazarrasa, Rosalind McAlpine, Francisco Moreno, Logan Neitzke-Spruill, Tehseen Noorani, Peter Oehen, Genís Ona, Fernanda Palhano-Fontes, Ryan Patchett-Marble, Gail Peekeekoot, Daniel Perkins, Katrin H. Preller, Johannes G. Ramaekers, Sara Reed, Dimitris Repantis, Brian Richards, William Richards, Sally Risby, Jessica Rochester, Ian Roullier, James Rucker, Simon Ruffell, James W. Sanders, Leonie Schneider, Thomas Shutte, Balázs Szigeti, Sayali Tadwalkar, Julien Thibault Lévesque, Christopher Timmermann, Keren Tzarfaty, Hattie Wells, Keith Williams

**Affiliations:** 1 Faculty of Medicine and Health Sciences, McGill University, Montreal, Quebec Canada; 2 Centre for Psychedelic Research, Department of Brain Sciences, Imperial College London, London, UK; 3 Lady Davis Institute for Medical Research, Jewish General Hospital, Montreal, Quebec Canada; 4 Department of Psychiatry, McGill University, Montreal, Quebec Canada; 5 Department of Psychology, University of Exeter, Exeter, UK; 6 Michigan Psychedelic Center, Department of Anesthesiology, University of Michigan, Ann Arbor, MI USA; 7 Yale School of Medicine, Yale University, New Haven, CT USA; 8 Psychedelic Research and Therapy Development, Adult Psychiatry and Psychotherapy, Psychiatric University Hospital Zurich, Zurich, Switzerland; 9 Clinical Research Center for Substance-Assisted Therapy, Division of Medicine, University of Basel, Basel, Switzerland; 10 International Center for Ethnobotanical Education Research and Services (ICEERS), Barcelona, Spain; 11 Psychedelic Research Lab, Monash University, Melbourne, Victoria Australia; 12 Brain Institute, Federal University of Rio Grande do Norte, Natal, Brazil; 13 Center for Psychedelic and Consciousness Research, Department of Psychiatry and Behavioral Sciences, Johns Hopkins University School of Medicine, Baltimore, MD USA; 14 Mood Disorders Psychopharmacology Unit, Toronto Western Hospital, University Health Network (UHN), Toronto, Ontario Canada; 15 Psychedelic Participant Advocacy Network (PsyPAN), London, UK; 16 Department of Psychiatry, University Medical Centre Groningen, Groningen, the Netherlands; 17 OPEN Foundation, Amsterdam, the Netherlands; 18 https://Breelove.ca; 19 Alma Viva Institute, São Paulo, Brazil; 20 Department of Psychiatry and Human Behavior, Warren Alpert Medical School of Brown University, Providence, RI USA; 21 Vancouver Island University, Nanaimo, British Columbia Canada; 22 Psychedelic Lived Experiences, Ottawa, Ontario Canada; 23 Center for Psychedelic Drug Research and Education, College of Social Work, Ohio State University, Columbus, OH USA; 24 University Writing Program, Johns Hopkins University, Baltimore, MD USA; 25 History of Health and Social Justice, University of Saskatchewan, Saskatoon, Saskatchewan Canada; 26 Department of Family Medicine, McGill University, Montreal, Quebec Canada; 27 Social Studies of Medicine, McGill University, Montreal, Quebec Canada; 28 Department of Psychiatry and Addictology, Faculty of Medicine, Université de Montréal, Montreal, Quebec Canada; 29 University of Florida Center for Arts in Medicine, Gainesville, FL USA; 30 Heffter Research Institute, Santa Fe, NM USA; 31 Columbia University Medical Center, New York State Psychiatric Institute, New York, NY USA; 32 Fluence, New York, NY USA; 33 NYU Grossman School of Medicine, New York University, New York, NY USA; 34 University Health Network Psychedelic Psychotherapy Research Group, University of Toronto, Toronto, Ontario Canada; 35 Graduate Program in Science, Technology and Society, Bar Ilan University, Ramat Gan, Israel; 36 Clinical Pharmacology and Toxicology, Department of Biomedicine and Department of Clinical Research, University Hospital Basel, Basel, Switzerland; 37 University of California, San Francisco, San Francisco, CA USA; 38 Birkbeck, University of London, London, UK; 39 The New School for Social Research, The New School, New York, NY USA; 40 International Laboratory for Brain, Music and Sound Research, Centre for Research on Brain, Language and Music (BRAMS-CRBLM), Montreal, Quebec Canada; 41 Michigan Psychedelic Center, University of Michigan, Ann Arbor, MI USA; 42 The Being Ground, Whitingham, VT USA; 43 Rory Meyers College of Nursing, New York University, New York, NY USA; 44 Psychology and Language Sciences, University College London, London, UK; 45 Department of Psychiatry, College of Medicine-Tucson, University of Arizona, Tucson, AZ USA; 46 Center for Medical Ethics and Health Policy, Baylor College of Medicine, Houston, TX USA; 47 School of Pharmacy, University of Auckland, Auckland, New Zealand; 48 Biberist, Switzerland; 49 Fundació Sant Joan de Déu, Hospital Sant Joan de Déu, Barcelona, Spain; 50 NOSM University, Thunder Bay, Ontario Canada; 51 Roots to Thrive, Nanaimo, British Columbia Canada; 52 Psychedelics Research and Therapeutics Unit, Melbourne School of Population and Global Health, University of Melbourne, Melbourne, Victoria Australia; 53 Department of Adult Psychiatry and Psychotherapy, Psychiatric University Clinic Zurich, University of Zurich, Zurich, Switzerland; 54 Faculty of Psychology and Neuroscience, Maastricht University, Maastricht, the Netherlands; 55 Psychedelic Substances Research Group, Department of Psychiatry and Neurosciences, Charité—Universitätsmedizin Berlin, Berlin, Germany; 56 Gladstone Psychiatry and Wellness, Baltimore, MD USA; 57 Sunstone Therapies, Rockville, USA; 58 Céu do Montréal, Montreal, Quebec Canada; 59 Department of Psychological Medicine, Institute of Psychiatry, Psychology and Neuroscience, King’s College London, London, UK; 60 South London and Maudsley NHS Foundation Trust, London, UK; 61 School of Population and Global Health, University of Melbourne, Melbourne, Victoria Australia; 62 DMT Research Group, Centre for Psychedelic Research, Department of Brain Sciences, Imperial College London, London, UK; 63 Hammersmith Medicines Research, London, UK; 64 Department of Psychiatry and Behavioral Sciences, Weill Institute for Neurosciences, University of California, San Francisco, San Francisco, CA USA; 65 MAPS Israel, Hod Hasharon, Israel; 66 Faculty of Humanities and Social Sciences, Centre for Interdisciplinary Studies, Athabasca University, Athabasca, Alberta Canada

**Keywords:** Clinical trial design, Psychology, Interdisciplinary studies, Research data, Clinical pharmacology

## Abstract

Psychedelic substances exhibit complex interactions with the ‘set and setting’ of use, that is, the mental state of the user and the environment in which a psychedelic experience takes place. Despite these contextual variables’ known importance, psychedelic research has lacked methodological rigor in reporting extra-pharmacological factors. This study aimed to generate consensus-based guidelines for reporting settings in psychedelic clinical research, according to an international group of psychedelic researchers, clinicians and past trial participants. We conducted a Delphi consensus study composed of four iterative rounds of quasi-anonymous online surveys. A total of 89 experts from 17 countries independently listed potentially important psychedelic setting variables. There were 770 responses, synthesized into 49 distinct items that were subsequently rated, debated and refined. The process yielded 30 extra-pharmacological variables reaching predefined consensus ratings:i.e., ‘important’ or ‘very important’ for ≥70% of experts. These items compose the Reporting of Setting in Psychedelic Clinical Trials (ReSPCT) guidelines, categorized into physical environment, dosing session procedure, therapeutic framework and protocol, and subjective experiences. Emergent findings reveal significant ambiguities in current conceptualizations of set and setting. The ReSPCT guidelines and accompanying explanatory document provide a new standard for the design and documentation of extra-pharmacological variables in psychedelic clinical research.

## Abstract (web summary)

A large international panel of experts reach consensus on a new standard for reporting settings in psychedelic trials, identifying 30 key non-pharmacological factors to strengthen the rigor of psychedelic research.

## Main

A guiding axiom of the psychedelic paradigm is that psychedelics’ pharmacological effects interact with extra-pharmacological variables in complex ways to produce their powerful effects on consciousness^1,2^. Extra-pharmacological contexts, which include physical environments, music, attitudes and social interactions, are typically dichotomized as ‘(mind)set and setting’^3^. This concept was popularized in the twentieth century to explain how psychedelic drug effects can range from psychotic like to mystical like, from the same drug at the same dose^3^.

Psychedelic drugs are currently subject to intensive study as potential psychiatric treatments^4,5^. Despite the critical influence of extra-pharmacological factors on these treatments, there is little known about their relative importance^6,7^, their scope^8^ or the temporality of their effects^9^. There is no consensus regarding which non-pharmacological factors most influence psychedelic drug effects.

These major knowledge gaps are perpetuated by inconsistent reporting practices. A recent systematic review of 33 clinical psychedelic studies found that many trials failed to report basic aspects of their contexts, including study locations and physical environments^10^. Such omissions limit the transparency and validity of psychedelic research^10–12^; if psychedelic drug effects are indeed the product of drug-context interactions, methodologically rigorous research requires clear and reliable reporting of contextual variables^13^.

The primary aim of this study was to develop guidelines for reporting extra-pharmacological variables in psychedelic clinical trials, initially focused on settings, as has been done for other complex health interventions^14–16^. We aimed to generate guidelines that were parsimonious, straightforward to implement and reflective of diverse perspectives. To achieve this objective, our study used the Delphi method, an evidence-based approach to deriving expert consensus on complex topics through iterative surveys^17^. This method was selected for its capacity to integrate diverse forms of academic and experiential knowledge^18^, to facilitate discourse across vast geographical regions^19^ and to enable the productive exchange of knowledge across power differentials and hierarchies by preserving anonymity^20^. To guide future research, the study also aimed to evaluate how set and setting are currently conceptualized in the field.

In this study, ‘experts’ were defined as people ‘having, involving, or displaying special skill or knowledge derived from training or lived experience’^21^. Experts were recruited among psychedelic clinicians, psychedelic researchers and former psychedelic clinical trial participants with significant knowledge or experience in at least one of the following related areas: psychopharmacology, neuroimaging, psychedelic-assisted therapy, clinical trial design and/or harm reduction. Expert panel diversity was established based on self-reported sociodemographic characteristics and professional affiliations.

This global Delphi study produced the Reporting of Setting in Psychedelic Clinical Trials (ReSPCT) guidelines, presented herein. Their 30 items represent the first international consensus regarding which specific non-pharmacological elements exert the most important influences on psychedelic drug effects, and thus warrant routine reporting in psychedelic clinical trials. These guidelines aim to significantly improve the quality of psychedelic research, much like other reporting guidelines have strengthened the evidence base of other clinical interventions.

## Results

### Recruitment and study flow

**Fig. 1:**
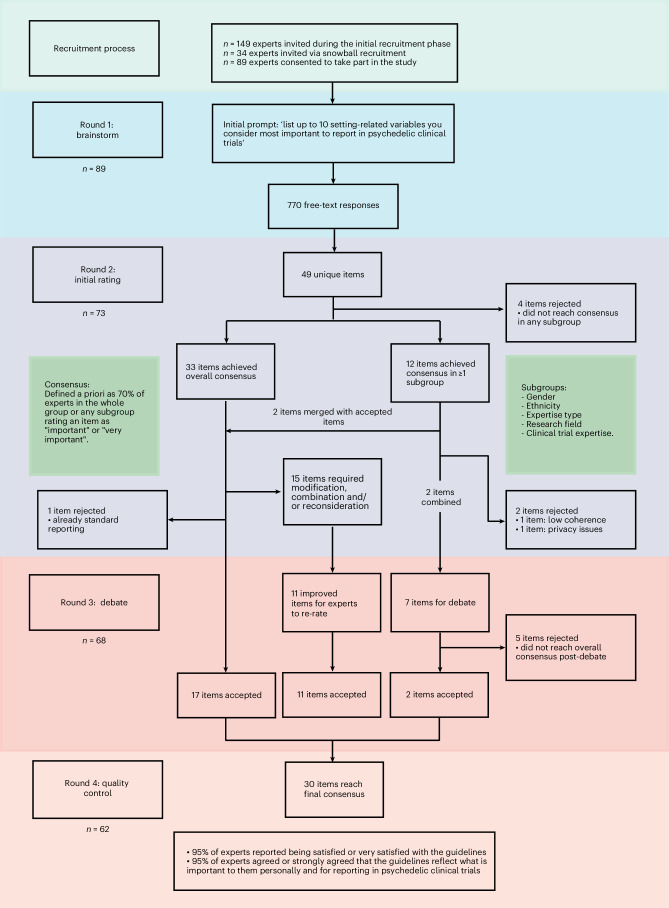
Flow diagram for study participants and items. Diagram showing the Delphi study flow, from recruitment to the final round. The diagram includes the number of participating experts per round, and the item’s progression from inception to being accepted, debated, improved or rejected.

Personalized email invitations to participate were initially sent to 149 experts, followed by an additional 34 experts identified by snowball recruitment. A total of 89 experts (48.6%) consented to participate, and all fully completed the first round. Thereafter, 73 experts completed round 2, 68 experts completed round 3 and 62 experts completed round 4, yielding a 30% attrition rate (Fig. 1).

### Demographics

The demographic and professional characteristics of the experts who completed the first and final rounds of this study are presented in Table 1, and affiliations are listed at the end of the article.

**Table 1:** Expert participants’ sociodemographic and professional characteristics in the first and final study rounds

Expert demographics		Round 1 (*n* = 89)		Round 4 (*n* = 62)	
	Category	Count	Percentage	Count	Percentage
**Gender identity**	Female	36	40.4%	26	41.9%
	Male	48	53.9%	32	51.6%
	Nonbinary	4	4.5%	3	4.8%
**Age**	25–34	19	21.3%	11	17.7%
	35–44	33	37.1%	25	40.3%
	45–54	21	23.6%	13	21.0%
	55–64	8	9.0%	7	11.3%
	65+	8	9.0%	6	9.7%
**Ethnicity**	Asian (Indian, Pakistani, Bangladeshi, Chinese, any other Asian background)	3	3.4%	2	3.2%
	Black, African or Caribbean	1	1.1%	0	0.0%
	Hispanic or Latino	1	1.1%	1	1.6%
	Indigenous	7	7.9%	4	6.5%
	Middle Eastern or North African	3	3.4%	1	1.6%
	Mediterranean	2	2.2%	1	1.6%
	Mixed two or more ethnic groups	5	5.6%	2	3.2%
	White	66	74.2%	50	80.6%
	Prefer not to say	1	1.1%	1	1.6%
**Nationality**	United States	22	24.7%	16	25.8%
	United Kingdom of Great Britain and Northern Ireland	17	19.1%	14	22.6%
	Canada	17	19.1%	12	19.4%
	Australia	5	5.6%	3	4.8%
	Switzerland	5	5.6%	4	6.5%
	Netherlands	4	4.5%	3	4.8%
	Brazil	3	3.4%	3	4.8%
	Germany	3	3.4%	0	0.0%
	Spain	3	3.4%	1	1.6%
	Denmark	2	2.2%	0	0.0%
	Israel	2	2.2%	1	1.6%
	Other: Chile, France, Greece, Hungary, Mexico or South Africa	6	6.7%	5	8.1%
**Education level**	Bachelor’s degree	8	9.1%	7	11.3%
	Master’s degree	18	20.5%	11	17.7%
	Doctorate or professional degree	59	67.0%	40	64.5%
	Vocational school	3	3.4%	3	4.8%
**Expert type**^**a**^	Researcher	64	71.9%	42	67.7%
	Clinician	39	43.8%	24	38.7%
	Participant	19	21.3%	16	25.8%
**Field of expertise**^**a**^	Science	62	69.7%	37	59.7%
	Psychology	42	47.2%	28	45.2%
	Social science (other than psychology)	44	49.4%	31	50.0%
	Plant medicine	4	4.5%	2	3.2%
	N/A	10	11.2%	9	14.5%
**Years working in psychedelic field**	1–5	26	29.2%	20	32.3%
	5–10	19	21.3%	11	17.7%
	10–15	12	13.5%	8	12.9%
	15+	32	36.0%	23	37.1%
**Self-reported form of psychedelic expertise**^**a**^	Lived experience	50	56.2%	41	66.1%
	Set and setting	46	51.7%	31	50.0%
	Psychedelic therapy in a clinical context	45	50.6%	33	53.2%
	Conducting clinical trials	30	33.7%	21	33.9%
	Psychedelic therapy in nonclinical contexts	27	30.3%	19	30.6%
	Psychedelic psychopharmacology	23	25.8%	15	24.2%
	Drug policy and/or harm reduction	21	23.6%	17	27.4%
	Psychedelic neuroimaging	14	15.7%	8	12.9%
	Other: physiotherapy, data science, ancestral medicine, design, literature, arts and esthetics, spiritual care, nursing or social work	13	14.6%	7	11.3%
**Self-reported expertise regarding specific psychedelic drugs**^**a**^	Psilocybin	80	89.9%	54	87.1%
	MDMA	52	58.4%	37	59.7%
	LSD	48	53.9%	33	53.2%
	Ayahuasca	40	44.9%	29	46.8%
	Ketamine	38	42.7%	29	46.8%
	DMT	33	37.1%	23	37.1%
	Mescaline	26	29.2%	20	32.3%
	5-MeO-DMT	18	20.2%	12	19.4%
	Ibogaine	5	5.6%	5	8.1%
	Other: cannabis, tobacco, salvinorin A, MDA, DBP, nitrous oxide	8	9.0%	5	8.1%

N/A, not applicable; 5-MeO-DMT, 5-methoxy-N,N-dimethyltryptamine; MDA, 3,4-methylenedioxyamphetamine; DBP, *N*,*N*-dipropyltryptamine

^a^Percentages do not amount to 100% as experts may have endorsed more than one form of expertise.

### Delphi round 1

Round 1 (*n* = 89) prompted experts to list up to ten variables that they consider most important to report in psychedelic clinical trials and to provide an optional explanation for their choices. This yielded 770 free-text responses and rationales, which often related to trial participants’ senses of safety and comfort under the influence of psychedelic drugs. These responses were then coded and grouped into a list of 49 unique items (Supplementary Table 2). Each item had been independently suggested by at least two experts, with four items mentioned by at least half of respondents: music and sound (*n* = 74); objects, decorations and artwork (*n* = 55); number of people present (*n* = 52); and access to nature (*n* = 46).

### Delphi round 2

In round 2 (*n* = 73), 33 of the 49 items (67%) from round 1 reached whole-group consensus thresholds. One of these, pertaining to aspects of the study protocol, was eliminated based on expert feedback that it is already subject to standard reporting practices in clinical trials, and 15 others were flagged for modification to address issues with coherence, uniqueness and/or implementability. At this stage, according to the study’s original aims, items that were identified as being largely subjective—that is, pertaining more to set than to setting—were flagged for implementability issues.

Of the 16 items not reaching whole-group consensus, 12 met the consensus threshold in one or more subgroups. Among them, one item was rejected for having a low coherence rating, and another was rejected because of potential privacy concerns for research study personnel. Two items were merged into items that had already reached whole-group consensus, and two other items were combined into a single item to address issues with overlap. The subgroup analyses thus yielded seven items to be debated in round 3.

Overall, during round 2, 17 items were accepted as written, 7 items were rejected and 27 items were combined into 18 based on whole- and/or subgroup feedback.

### Delphi round 3

In round 3 (*n* = 68), all newly modified items met the 90% coherence threshold, and the proposed combinations of seven items into three new items surpassed 90% acceptance. Re-ratings of the seven debated items, which had previously reached only subgroup consensus (Supplementary Table 3), resulted in two items reaching whole-group consensus and thus being incorporated in the final guidelines (Table 2).

**Table 2:** Items debated in round 3 due to reaching consensus threshold in one or more subgroups but not the overall group

Debated item	Ratings in the overall group	Ratings in groups in which the item met consensus threshold	Reasons provided for (and against) the item’s inclusion in reporting guidelines
**Items that ultimately achieved consensus**			
Bathroom facilities	60%	BIPOC or mixed ethnicity: 14/18 (78%) Past trial participants: 16/20 (80%)	Bathroom accessibility and privacy may influence the experiences of participants in vulnerable states induced by psychedelic drugs.
Facilitators’ cultural competence and safety^a^	52% competence 47% safety	BIPOC or mixed ethnicity: 14/18 (78%) competence 13/18 (72%) safety Plant medicine: 4/4 (100%) both Nonbinary: 3/4 (75%) safety	Study facilitators’ capacity to provide culturally competent and safe care may impact how participants from diverse backgrounds experience the study, and have important ramifications on the broader field’s diversity, equity and inclusivity.
**Items that ultimately failed to achieve consensus**			
Odors, scents, smells	58%	Past trial participants: 15/20 (75%) Plant medicine: 4/4 (100%)	The capacity for olfactory stimuli to invoke memories and/or influence emotions may be heightened under the effects of psychedelic drugs.
Temperature	58%	BIPOC or mixed ethnicity: 15/18 (83%) Past trial participants: 14/20 (70%) Social sciences: 19/26 (73%) Plant medicine: 4/4 (100%)	Temperature may influence participant comfort and sense of control over their environment, and thereby shape their psychedelic experience.
Facilitators’ demographics and cultural backgrounds	60%	Nonbinary: 3/4 (75%) BIPOC or mixed ethnicity: 14/18 (78%) Clinicians: 22/31 (71%) No clinical trial experience: 19/25 (76%)	Study facilitators’ backgrounds and beliefs may directly or indirectly shape psychedelic experiences through interactions with participants. However, collecting and reporting such information may raise privacy concerns.
Sociocultural context	56%	Nonbinary: 3/4 (75%) BIPOC or mixed ethnicity: 15/18 (83%) Past trial participants: 14/20 (70%) Plant medicine: 4/4 (100%)	Psychedelic experiences may be influenced by both current and past societal factors, including media coverage, legal statuses and mainstream perceptions of psychedelic drugs. However, such influences may be too broad and complex to be accurately captured and reported.
Social determinants of health	68%	Male: 27/38 (71%) Nonbinary: 3/4 (75%) BIPOC or mixed ethnicity: 16/18 (89%) Clinicians: 23/31 (74%) Social sciences: 22/26 (85%) Plant medicine: 4/4 (100%) No clinical trial experience: 19/25 (76%)	Social determinants of health exert important influences on mental and physical well-being, and probably influence psychedelic clinical trial outcomes. However, social determinants are defined in diverse ways, and they may be challenging to accurately report owing to their breadth and complexity.

^a^Cultural competence and cultural safety were originally two separate items that were merged as per expert feedback. BIPOC, Black, Indigenous, People of Color.

Experts were also asked to vote regarding the potential inclusion of the five items collectively identified as referring more to set than to setting owing to their subjectivity. Despite the study’s initial focus on setting variables, only 15% of experts voted to exclude these items from the guidelines. Rather, experts supported keeping these items as a standard component of the guidelines (46%) or as an optional subsection pertaining to ‘participant experience’ (39%). On the basis of these responses, these five items examining subjective experiences were included in the final section of the guidelines (Table 3).

**Table 3:** ReSPCT 2025 guidelines

Item	Item number	Item details
**Physical environment**		
Location	1	Location of the trial, whether indoors or outdoors and urban, rural or suburban
Ambiance	2	Room ambiance curated by the study team
Access to nature	3	Sources of nature or natural elements that are physically or visually accessible to participants
Objects and decorations	4	Objects and decorations in the room
Lighting	5	Room lighting and adjustability
Sensory reduction	6	Sensory reduction used, such as headphones and eyeshades
Bathroom facilities	7	Level of bathroom accessibility, privacy and safety
**Dosing session procedure**		
Number and roles of people present	8	Number and roles of people present, including participants, study staff and informal support
Positioning	9	Relative position of people in the room and what participants were positioned on (for example, bed, mat, couch)
Focus and main activities	10	Focus (internal or external) and main activities of the dosing session
Music or soundscapes	11	Music or soundscapes that accompanied the dosing experience
Interpersonal interventions	12	Verbal or physical interpersonal interventions used throughout the session and how consent was obtained
Participant autonomy, control and agency	13	Level of participant control and agency over activities and environment of the dosing session
Dosing regimen	14	Dosing regimen, including drug dose(s), frequency, route of administration and length of the dosing session
Medical and experimental procedures or assessments	15	Medical and experimental procedures or assessments performed during the dosing session
Pre- and post-dosing protocol	16	Activities that took place immediately before or after dosing, including participant arrival and release conditions
Potential disturbances or interruptions	17	Disturbances or interruptions that may have impacted the quality of the dosing session
**Therapeutic framework and protocol**		
Therapeutic or guiding approach	18	Therapeutic or guiding approach used throughout the study, if any, with the accompanying manual or protocol
Narrative framing	19	Framing of the trial intervention by the study team, including the short- and long-term drug effects
Number of sessions	20	Number and length of preparation, dosing and integration sessions
Preparation protocol	21	Activities performed during the preparation sessions
Integration protocol	22	Activities performed during the integration sessions
Additional support or follow-up	23	Formal or informal support or follow-up offered to participants after the end of the trial, including in the case of adverse events
Study personnel qualifications	24	The credentials, training and expertise of personnel providing the study intervention or care
Cultural competence and safety	25	Study team’s level of cultural competence and efforts toward cultural safety
**Subjective experiences**		
Therapeutic alliance	26	Therapeutic alliance between participants and facilitators throughout the intervention
Trust	27	Participant’s level of trust throughout the intervention
Physical comfort	28	Participant’s level of physical comfort during the dosing session
Physical safety	29	Participant’s sense of physical safety during the dosing session
Psychological and cultural safety	30	Participant’s sense of psychological and cultural safety with the people present during the dosing session

The ReSPCT guidelines serve as a reporting tool for settings in clinical trials with psychedelics. Each item should be reported in the main text or supplement of the resultant publication(s), the location of which should be indicated in the accompanying checklist (Supplement 1 in the Supplementary Information). The supporting explanatory document (Supplement 2 in the Supplementary Information) provides details and further guidance, including relevant research and suggested reporting prompts for each item. All documents can also be found on the ReSPCT website (https://respctguidelines.com/).

Overall, round 3 resulted in 13 additional items meeting criteria for acceptance. Added to the 17 accepted items from round 2, this produced the 30-item ‘Preliminary guidelines for reporting setting in psychedelic clinical trials’ (Supplementary Table 4) with an overview of each items’ progression from round 1 to round 4 (Supplementary Table 5).

### Delphi round 4

Results from the final round (*n* = 62) are presented in full in Supplementary Table 6. At least 95% of responding experts reported being satisfied or very satisfied with the preliminary guidelines, and either agreed or strongly agreed that they reflect what is important for reporting in psychedelic clinical trials. At least 74% of responding experts said they would ‘definitely’ use the preliminary guidelines, and 25% said they would ‘consider it’; only 1 (2%) expert reported they would not. Feedback provided from the study experts expressing hesitation about using the preliminary guidelines (summarized in Supplementary Table 7) informed further improvements, which subsequently obtained approval from the study experts and culminated in the final guidelines presented below.

Regarding the study methodology and process, at least 95% of experts rated the survey instructions as clear, the study structure as generally satisfactory, and both deadlines and time commitments as reasonable. Similarly, at least 95% evaluated the study as successful in identifying areas of agreement and disagreement, allowing opinions to be comfortably expressed, and ensuring that diverse voices had been adequately heard. Finally, 68% of experts reported that their conceptualization of setting had changed as a result of the study process, mostly in terms of the granularity, scope and depth with which they considered setting-related variables. The rates of reported changes in conceptualization did not differ between subgroups or geographical regions.

### The ReSPCT guidelines

The final ReSPCT 2025 guidelines are presented in Table 3 (fillable format in Supplement 1 in the Supplementary Information) and available on the ReSPCT website (https://respctguidelines.com). They were collaboratively refined from the preliminary guidelines by the study leads and experts. The guidelines are accompanied by a comprehensive explanatory document (Supplement 2 in the Supplementary Information), also produced collaboratively, which provides detailed descriptions of each item, suggested reporting prompts, supporting expert responses and a brief summary of the literature on the 30 ReSPCT items.

The ReSPCT guidelines are composed of 30 items divided into 4 sections. The first section describes the physical and sensory environments to which participants are exposed during a dosing session, such as the study location, decorations and sensory reduction devices available to participants. The second section pertains to the dosing procedure, including the people present, music provided and interventions performed. The third section regards the trial’s broader therapeutic framework and protocol, including preparatory and integration activities^1^, and study personnel qualifications. The final section explores key aspects of participants’ subjective experiences, including evaluations of therapeutic alliances, comfort, and both physical and psychological safety.

### The conceptualization of set and setting

**Fig. 2:**
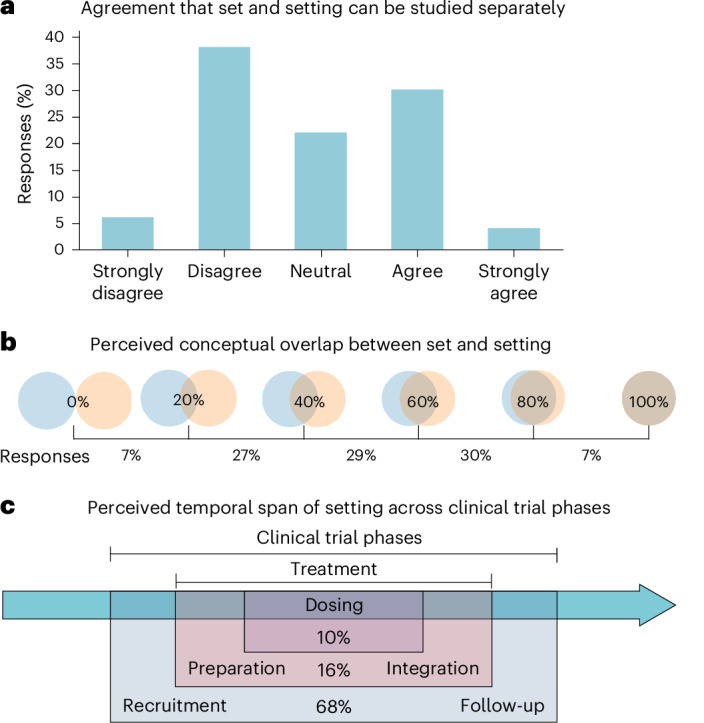
Expert responses regarding the separability and conceptual overlap of set and setting factors, and temporal span of setting in psychedelic clinical trials. **a**, Experts rated their level of agreement with the statement ‘set and setting can be studied separately’ on a 5-point Likert scale. **b**, Experts rated the conceptual overlap between set and setting on a scale from 0 (no overlap) to 100 (complete overlap). **c**, Experts indicated the span of setting in the context of a clinical trial according to the following options: from the start to the end of the dosing session, throughout the treatment phase (preparation, dosing and integration), from recruitment to follow-up or other.

As detailed above, an emergent finding of the study’s first three rounds was a lack of clarity and consensus regarding the conceptual boundaries between ‘set’ and ‘setting’. Additional questions were thus posed to experts in round 4 to explore perspectives on this influential concept (Fig. 2). When asked to rate their agreement with the statement that set and setting can be studied separately, almost equal proportions agreed or strongly agreed (37%) and disagreed or strongly disagreed (42%). Experts were also asked to estimate the conceptual overlap between set and setting, from 0% to 100%. Responses again ranged widely, with nearly equal proportions of experts estimating conceptual overlap as 20–40%, 40–60% or 60–80%, yielding an average overlap rating of 49%.

In addition, experts were asked to define the temporal boundaries of setting across the standard phases of psychedelic clinical trials. About 68% agreed that setting spans from the start of a clinical trial’s recruitment to the end of its follow-up. Only 16% considered setting as limited to the trial’s treatment phase—generally entailing preparation, dosing and integration subphases^1^—and 10% considered setting to be confined to the actual dosing session.

## Discussion

### The ReSPCT guidelines

The 30-item ReSPCT guidelines provide a new standard for the reporting of extra-pharmacological variables in psychedelic clinical trials, derived from an international Delphi consensus study of experts from 17 different countries and 50 different institutions. The contributing clinicians, researchers and past trial participants were selected to represent diverse forms of knowledge and perspectives, which were exchanged and debated throughout the iterative Delphi process. The final items span the physical environment of dosing sessions to important aspects of participants’ experiences of psychedelic clinical trial settings. The guidelines, downloadable checklist (Supplement 1 in the Supplementary Information) and explanatory document (Supplement 2 in the Supplementary Information) can be found on the ReSPCT Guideline website (https://respctguidelines.com).

The items of the ReSPCT guidelines are intended to be reported in the texts or supplements of future studies alongside a completed checklist (Supplement 1 in the Supplementary Information). In the case of multisite clinical trials, we recommend completing the whole guidelines based on the overarching study protocol and setting, and also specifying distinct features of each site by, for instance, including photographs of each treatment room. This practice aims to strengthen the design and documentation of clinical research on psychedelic drugs and thereby improve the transparency, reproducibility and validity of this rapidly evolving field^12,22^. The adoption of these guidelines may also facilitate data comparison and aggregation across different groups and institutions^23^, and inform future dismantling and therapeutic studies^6^. Indeed, such issues recently prompted the US Food and Drug Administration to describe psychedelic therapies as a ‘black box’^24^ and contributed to their recent rejection of 3,4-methylenedioxymethamphetamine (MDMA)-assisted therapy as a treatment for post-traumatic stress disorder^25^.

The ReSPCT guidelines were designed specifically for psychedelic clinical trials but may be adapted to other clinical and research contexts. For instance, they may be valuable in research involving other acutely psychoactive substances, given that potent drug-context interactions have been found with multiple non-psychedelic substances—including alcohol, antidepressants and opiates^11^. In addition, the guidelines may facilitate more observational or pragmatic forms for psychedelic research^26^. The challenges of blinding in psychedelic research may prove to be insurmountable^27^; the ReSPCT guidelines could therefore support pragmatic study designs that prioritize the careful curation and systematic reporting of contextual variables over efforts to mask or minimize their influences^26,28^.

### Heterogenous views on set and setting

The study revealed diverse initial views regarding the relative importance of various components of settings. Some of the final items, such as music and access to nature, were independently suggested by most experts in the first round and retained high agreement throughout the study. Others, such as narrative framings, and the positioning of people in treatment rooms, were mentioned by few but rapidly reached consensus thresholds thereafter.

Two items that feature in the ReSPCT guidelines initially failed to reach whole-group consensus but were rescued by the debate process. For instance, in round 2, ‘Cultural competence and safety’ (item 25) reached consensus only in two subgroups. Cultural competence refers to a study team’s ability to care for participants with diverse values, beliefs and behaviors^29^, while cultural safety focuses on increasing health equity by encouraging healthcare professionals to examine power imbalances in patient-provider interactions^30^. In round 3, multiple experts supported this item’s importance, citing concerns regarding power imbalances and a lack of diversity in psychedelic clinical and research contexts^31–34^. As a result, the item’s whole-group rating increased from 50% to 73%, leading to its inclusion in the final guidelines.

The study process also identified challenges associated with how set and setting is currently conceptualized. Despite the study’s original focus on setting variables, early results and a subsequent vote led to the inclusion of five items that align more closely with the concept of (mind)set in the final guidelines’ last section. In contrast to the consensus achieved regarding the relative importance of specific extra-pharmacological variables, expert views on the concept of set and setting itself remained highly heterogenous. For instance, in the final round, expert views were evenly divided regarding the perceived overlap and the separability of set and setting variables.

The discrepant understandings of ‘set and setting’ among experts probably contributes to inconsistent reporting practices in psychedelic clinical trials. The ReSPCT guidelines aim to bypass this confusion with an explicit list of 30 key extra-pharmacological factors that are understood as interrelated, rather than as dichotomous. That is, a study’s environment and therapeutic approach may interactively shape participant experiences, and those experiences may influence how extra-pharmacological factors are perceived or even adapted. The guidelines thus prioritize specificity over simplicity in the aim of improving psychedelic clinical research practices. Further study is needed to evaluate whether the utility of ‘set and setting’ outweighs its conceptual confusion in other contexts.

Despite the study’s strengths, several limitations must be acknowledged. First, the Delphi process was entirely survey based, which may have reduced the spontaneity and depth of dialog that can occur in face-to-face interactions. Second, the study’s sample of experts did not represent the full diversity of perspectives on these drugs. For instance, the study’s focus on clinical trials excluded broader, nonclinical perspectives on variables that shape psychedelic experiences. It also resulted in most experts being relatively young and originating from Western countries, where psychedelic clinical research is currently most highly concentrated. Relatedly, the study’s definition of expertise reproduced known issues of the psychedelic field^32,33^, namely, the current lack of racial diversity among psychedelic researchers and trial participants. This limitation was only partially overcome through the study’s subgroup process, given that 75–80% of the sample was White. Finally, expertise does not necessarily equate to truth, and opinions of current psychedelic experts must be situated in the current historical moment and body of knowledge. Although this study intentionally enrolled experts with diverse views on psychedelic drugs, there was general agreement that psychedelic experiences and the contextual factors that shape them are clinically and scientifically important. The study did not include, for instance, experts who suggest that psychedelic drug experiences may be more akin to epiphenomena^35,36^, which may have influenced the study’s eventual findings. Further empirical research is needed to evaluate the actual importance of the variables that have been included in, or excluded from, the ReSPCT guidelines, and to discern those most relevant to clinical outcomes.

The ReSPCT guidelines aim to advance the study of psychedelic drugs and therapies just as reporting guidelines have improved the transparency and interpretability of other complex clinical interventions^15,16,37^. Its 30 items reflect a necessary, if preliminary, expert consensus regarding the most important contextual factors to report in studies of psychedelic drugs. The accompanying explanatory document serves to facilitate reporting and guide future research by synthesizing the current literature regarding contextual influences on psychedelic drug effects. The most up-to-date version of the ReSPCT guidelines and associated documents can be found at https://respctguidelines.com/. Routine utilization of these guidelines could significantly improve the rigor of psychedelic research, mitigate the ambiguities of current understandings of set and setting, advance our understanding of drug-context interactions, and potentially facilitate the future clinical implementation of these promising therapies in safe, transparent and effective ways.

## Methods

The definition of ‘psychedelics’ varies but is herein broadly defined to include the classical serotonergic psychedelics such as psilocybin, lysergic acid diethylamide (LSD) and dimethyltryptamine (DMT)^38^; ‘atypical’ psychedelics such as ibogaine and salvinorin A; and other acutely psychoactive drugs used as adjuncts to psychotherapy, such as MDMA and ketamine^39^.

This project was designed and implemented according to the Conducting and Reporting Delphi Studies standards (Supplementary Table 8)^40^, in addition to other Delphi-specific guidelines available in the literature^18,41–43^. The study consisted of four sequential rounds of surveys, administered electronically in English using the digital platform Qualtrics, from April to August 2023.

The first round consisted of an open-ended question, while subsequent rounds solicited quantitative and qualitative expert feedback on the results of previous rounds. Free-text comments were solicited in each round. Consensus, our primary outcome, was defined a priori as a variable (or ‘item’) being rated as ‘important’ or ‘very important’ for inclusion in reporting guidelines by at least 70% of experts, on a 7-point Likert scale. The 70% threshold was selected as a reasonable intermediate between cut-offs used in similar Delphi studies^44^, which can range between 51% and 97% (ref. ^45^).

Summary statistics and qualitative analyses were conducted using Microsoft Excel, and plots were generated in both Excel and R (RStudio version 2021.09.0 + 351).

### Expert recruitment

Clinicians and researchers were identified through searches of the academic literature, conference websites, psychedelic-related institutes or organizations, and the authors’ academic networks. Snowball sampling was also used after initial invitations were sent^46^. Expertise was validated based on academic and professional qualifications, years of relevant experience and contributions to the field. Past trial participants were primarily recruited in collaboration with the cofounders of the Psychedelic Participant Advocacy Network (PsyPAN), a nonprofit organization based in the United Kingdom. The PsyPAN cofounders informed members about the study, and those who showed interest were put into contact with the study leads. Expert panel diversity was established based on self-reported sociodemographic characteristics and professional affiliations.

Although there is no standard sample size for Delphi studies, most include up to 25 participants^45^, and guidelines recommend at least 15–20 experts in any given subgroup for statistical purposes^47^. Accordingly, we aimed to recruit groups of 20 researchers, clinicians and past trial participants, for a total of 60 experts by round 4. Taking into account attrition, we sought enrollment of 90 experts for round 1 (ref. ^48^). All experts were recruited by email invitation, with a maximum of two reminders sent for each round. No new experts were sought once round 1 was completed (May 2023).

All participating experts were given the option to contribute as coauthors or be acknowledged in future publications, and former trial participants were additionally remunerated at a rate of £20 per completed survey, based on consultation with PsyPAN. This renumeration was supported with funding from the Imperial College London Societal Engagement Seed Fund, which supports patient and public involvement in research.

### Ethics

Ethical approval for this study was granted by the Imperial College Research Ethics Committee (reference number 6568054). All study participants provided online written consent at the beginning of the first survey. During data collection, participants were quasi-anonymous, that is, anonymous to other experts but identifiable to the study team^43^. After data collection, personal identifiers were replaced with unique participant identification numbers, with the identifying key encrypted and stored separately.

### Delphi round 1

The first survey underwent pilot acceptability and fidelity testing with six independent experts, including the two cofounders of PsyPAN. In round 1, all experts were asked to list up to ten setting variables they deemed most important for reporting in psychedelic clinical trials, with the option to provide their rationale for each. The lead author (C.P.-M.) first coded all free-text responses in a nonhierarchical manner. Subsequently, the three lead authors collaboratively grouped these codes into broader themes and iteratively refined them to improve clarity and reduce redundancy. This process yielded a preliminary list of distinct items, organized into preliminary sections, with item descriptions summarizing the details and rationales provided by experts.

### Delphi round 2

In round 2, experts were provided with the list of items and descriptions generated in round 1. For each item, experts were asked to rate the importance of its inclusion in the reporting guidelines and its coherence, that is, the clarity of its formulation and description, and to optionally provide additional feedback.

Importance ratings were analyzed according to the consensus threshold, both for the entire group and within predefined subgroups of experts according to gender, ethnicity, primary type of expertise, field of expertise and previous clinical trial experience (Supplementary Table 1). Subgroup analyses are not universal in Delphi studies^47^, but were conducted to identify minority opinions that may otherwise have been overlooked.

Items that reached consensus in the entire group or in any subgroup then underwent quality analyses based on coherence, uniqueness and implementability. Coherence was based on experts responding ‘yes’, ‘no’ or ‘unsure’ to the prompt ‘I think this item is coherent/logical’. Uniqueness and implementability were established based on solicited free-text feedback. Items with less than 90% coherence ratings were modified according to expert feedback; items lacking uniqueness were combined with other overlapping items; items identified as having potential implementability issues were flagged for reconsideration.

### Delphi round 3

In round 3, experts were asked to reevaluate the items that had reached consensus but had issues with coherence, uniqueness and/or implementability. For items modified to address coherence issues, experts re-rated the modified item’s importance and coherence. For items combined to reduce excessive overlap, experts indicated whether they agreed or disagreed with the proposed combinations. For items with identified implementability issues, experts voted whether to keep them in the core guidelines, move them to a separate optional subsection or remove them altogether.

Experts were also asked to debate items that reached consensus in at least one subgroup but not across the full sample. They were presented with each debated item’s importance scores for the entire group and for the subgroup(s) in which the item reached consensus, alongside representative quotes spanning the divergent opinions. They then re-rated the debated item’s importance based on this additional information.

Lastly, experts were presented with a table of items that did not reach consensus in any subgroup and invited to voice any final opposition to their rejection from the guidelines.

### Delphi round 4

In round 4, experts were asked to provide feedback on the preliminary reporting guidelines developed over the previous three rounds and to rate the perceived quality of the study process. In addition, based on emergent findings of the previous rounds, experts were asked to rate the conceptual overlap, separability and temporal boundaries of set and setting, and to report whether their understanding of this concept had evolved throughout the study. Quantitative feedback was solicited using various 5-point Likert scales, and qualitative feedback was collected via free-text responses.

### Reporting summary

Further information on research design is available in the Nature Portfolio Reporting Summary linked to this article.

## Online content

Any methods, additional references, Nature Portfolio reporting summaries, source data, extended data, supplementary information, acknowledgements, peer review information; details of author contributions and competing interests; and statements of data and code availability are available at 10.1038/s41591-025-03685-9.

## Supplementary information

Supplementary Information Supplementary Tables 1–8, Supplement 1 (fillable checklist) and Supplement 2 (explanatory document).

Reporting Summary

## Data Availability

Given the partially anonymous nature of Delphi studies and the personal opinions shared by participants, data will not be deposited in a public repository. Rather, deidentified individual-level data, along with additional study materials such as study surveys, will be made available upon reasonable request for a minimum of 5 years following publication. Requests should be directed to corresponding author C.P.-M. and include the following information: a brief proposal including study aims, data requested, planned analysis and credentials of the requestor(s). All requests will be reviewed by the study lead authors based on scientific merit, feasibility and ethical considerations. An initial response will be provided within 2 weeks. For accepted proposals, data sharing will be contingent on an executed data transfer agreement.
