# Supplementary material for: An international Delphi consensus for reporting of setting in psychedelic clinical trials
Source: Nat Med. 2025 Jun 3;31(7):2186–95. doi: 10.1038/s41591-025-03685-9 (PMC12283393; doi:10.1038/s41591-025-03685-9)
Supplement: Supplementary file 1 — Supplementary Tables 1–8, Supplement 1 (fillable checklist) and Supplement 2 (explanatory document). [file 41591_2025_3685_MOESM1_ESM.pdf]

nature medicine

Supplementary information

https://doi.org/10.1038/s41591-025-03685-9

An international Delphi consensus for reporting of setting in psychedelic clinical trials

In the format provided by the authors and unedited

Supplementary Information

An international Delphi consensus for reporting of Setting in Psychedelic Clinical Trials

Pronovost-Morgan, C., Greenway, K.T., Roseman, L., & The ReSPCT Experts:

[Aday, J.S., Agin-Liebes, G., Ahmad, K., Aicher, H., Aixalà, M., Amar, S., Araujo, D., Barrett, F.S., Belser, A., Blainey, M., Bowman, R., Bourne, M., Breeksema, J., Breelove Carter, K., Bussey, J., Camara, C., Canby, N., Chalchi, J., Close, J., Cox, T., Daraeizadeh, P., Davis, A., de la Salle, S., Devenot, N., Dyck, E., Erritzoe, D., Farzin, H., Friesen, P., Garel, N., Giblin, J., Golden, T., Greer, G., Gukasyan, N., Guss, J., Hapke, E., Hartogsohn, I., Holze, F., Kettner, H., Knowles, A., Langlitz, N., Lehmann, A., Lifshitz, M., Lucas, P., MacLean, K., Mallon, O., Marcus, O., Mazarrasa, J., McAlpine, R., Moreno, F., Neitzke-Spruill, L., Noorani, T., Oehen, P., Ona, G., Palhano-Fontes, F., Patchett-Marble, R., Peekeekoot, G., Perkins, D., Preller, K.H., Ramaekers, J.G., Reed, S., Repantis, D., Richards, B., Richards, W., Risby, S., Rochester, J., Roullier, I., Rucker, J., Ruffell, S., Sanders, J.W., Schneider, L., Shutte, T., Szigeti, B., Tadwalkar, S., Tzarfaty, K., Thibault Lévesque, J., Timmermann, C., Wells, H., Williams, K.]

Table of Contents

Supplementary Tables ... *2*

Supplementary Table 1. Pre-defined subgroups for Round Two analyses. ... 2

Supplementary Table 2. Round One table of items ... 3

Supplementary Table 3. Round Two subgroup analyses. ... 6

Supplementary Table 4. Preliminary guidelines presented to experts in Round Four. ... 9

Supplementary Table 5. Progression of items from Round 1 to Round 3, and corresponding item name in final guidelines. ... 11

Supplementary Table 6. Round Four results. ... 13

Supplementary Table 7. Concerns raised by experts regarding the preliminary guidelines. ... 14

Supplementary Table 8. Completed Pre-Recommendations for the Conducting and REporting of DElphi Studies (CREDES) Checklist ... 15

Supplement 1 ... *16*

ReSPCT Guidelines 2025 – Reporting Tabl ... 16

Supplement 2 ... *18*

ReSPCT Guidelines 2025 – Explanatory Document ... 18

Supplementary Tables

Supplementary Table 1. Pre-defined subgroups for Round Two analyses.

Caption: *BIPOC stands for 'Black, Indigenous, People of Color'. The 'BIPOC or mixed ethnicities' subgroup includes people who self-identified as Asian (Indian, Pakistani, Bangladeshi, Chinese, or any other Asian background), Black / African / Caribbean, Hispanic / Latino, Indigenous, Middle Eastern or North African, Mediterranean, or Mixed two or more ethnicities.

Sample characteristic	Subgroups
**Gender**	Female
	Male
	Non-binary
**Ethnicity**	White
	BIPOC or mixed ethnicities*
**Expert Type**	Past trial participant
	Researcher
	Clinician
**Field**	Biological
	Psychological
	Social
	Plant medicine
**Clinical Trial Experience**	Yes
	No

Supplementary Table 2. Round One table of items

Caption: Table of items generated from expert responses in Round One to the prompt "list up to 10 setting variables you consider most important to report in psychedelic clinical trials". In Round Two, experts were asking to rate each item in this table in terms of perceived importance and coherence, and provide option free-text comments.

**DOSING ROOM (1): Physical Environment**			
**#**	**Item**	**Frequency**	**Item details**
(1)	Objects / decorations / artworks	55	Types: religious / spiritual / personal / grounding / ceremonial / psychedelic / neutral<br>Examples: "altar", books, candles, photographs, statues, effigies, (wall) colors
(2)	Nature	45	Physical access (Y/N), visual access via window(s) (Y/N)<br>Living flowers / plants, other natural / nature-inspired elements
(3)	Facilities	36	Waiting room: "feel", level of comfort<br>Bathroom: accessibility, privacy, design<br>Kitchen or availability of food and drink
(4)	Location	33	Indoors vs. outdoors<br>Place: clinic / hospital / retreat centre / university / home / nature, etc.<br>Surroundings: e.g., urban / rural
(5)	Rest area	21	Type: bed / sofa / mattress / rug<br>Accessories: blankets, pillows<br>Body position: sitting / lying / reclining / standing
(6)	Layout / positioning	15	Of furniture / furnishings: tables, chairs, mirror (avoid), (salt) lamps, rugs, etc.<br>Of people present: facilitators relative to participant(s); if group, participants configuration (circle, line, etc.)
(7)	Equipment	4	Medical equipment: emesis bag, heart / BP monitors, etc.<br>Experimental equipment: MRI, EEG, filming / recording devices (ostentiveness), etc.
**DOSING ROOM (2): Sensory Stimuli**			
**#**	**Item**	**Frequency**	**Item details**
(8)	Lighting	37	Soft / harsh, natural / artificial, bright / dim / dark, tones
(9)	Global "feel"	28	Medical / sterile, neutral, spiritual, shamanic, homey / "living-room like"<br>Aesthetically pleasing / beautiful; messy vs. tidy
(10)	Sensory reduction devices	24	Eyeshades: type, quality<br>Earplugs or noise-cancelling headphones
(11)	Acoustics	23	Level of quietness / sound insulation<br>Auditory disturbances inside and outside the room: machines beeping, people talking, traffic noise, etc.
(12)	Odors / scents / smells	18	Intentional: essential oils, aromatherapy, other<br>Unintentional: sterile / chemical smells, other unpleasant smells
(13)	Temperature	16	Ambient temperature<br>Means to regulate participant temperature: fans, participant clothing (layers), blankets, etc.
(14)	Other	6	Textures (soft / smooth vs. rough / synthetic), electromagnetic and radio-frequency radiation, humidity, airflow
**DOSING SESSION(S) (1): Protocol**			
**#**	**Item**	**Frequency**	**Item details**
(15)	Music / other soundscapes	74	Music vs. soundtrack vs. voice (e.g. mantras, chants) vs. silence<br>Type/genre; playlist; narrative; lyrics (Y/N); rationale; expected role<br>Continuous vs. intermittent; in relation to drug PK and trip/process<br>Equipment / who can hear it (headphones, speakers); sound quality; volume
(16)	Participant autonomy / control / agency	40	Does the participant have any control over:<br>Behaviours (e.g., movements like walking or dancing, body position, changing locations);<br>Environment (e.g. bringing personal objects, input in room design);<br>Sensory stimuli (e.g., eyemask on/off, headphones on/off, adjusting lighting or temperature);<br>Music (y/n, loudness, playlist, genre).
(17)	Supportive interventions	25	Type: physical (touch, hand-holding, body work), verbal (check-ins, instructions, guidance), non-verbal (supportive silence)<br>Level / frequency: minimal to intensive
(18)	Disturbances / interruptions	20	Lack of privacy (from the outside world)<br>Time or space constraints (rushed process or overcrowded room)<br>Other distractions
(19)	Approach	17	Structured vs. flexible; standardized vs. personalized<br>Misc.: trauma-informed; patient-centered; non-judgmental; serious / reverential vs. light-hearted / use of humor
(20)	Procedures (if applicable)	9	Medical: blood draws, PK samples, heart monitoring, etc.<br>Experimental: intensity ratings, psychometric tests, experimental tasks, neuroimaging, interviews, etc.
(21)	Pre / post-dosing protocol	5	Pre: spoken / written outline of the day; introduction to objects that may be used during the session (e.g. headphones, weighted blanket); opening ritual, etc.<br>Post: closing ritual; debriefing, etc.

(22)	Dosing schedule	4	Drug(s) dose and time(s) of dosing (morning, afternoon, evening, night)<br>Length of the session and breaks
(23)	Process / main activities	2	Inwards: e.g., resting eyes-closed<br>Outwards: e.g., conversations/sharing, singing, dancing, chanting, walking through nature

DOSING SESSION(S) (2): People present

#	Item	Frequency	Item details
(24)	Who and how many?	52	Participant(s): individual vs. group<br>Formal support (study team): medical staff, research staff, facilitators<br>Informal support: loved ones (friends, family), peers
(25)	(Therapeutic) alliance	24	Therapeutic alliance between participant and each facilitator;<br>Alliance / interpersonal dynamics among participants and between the (two) facilitators
(26)	Study team professional backgrounds and skillsets	18	Qualifications, (psycho)therapeutic / academic background, level of expertise with psychedelics / altered-states of consciousness
(27)	Facilitators and other participant(s) demographics / cultural backgrounds	14	Gender, age, nationality, race / ethnicity, native language, sexual orientation, religious orientation / spiritual orientation / metaphysical beliefs
(28)	Study team mindsets	9	Prevalent attitudes (e.g. level of confidence) and intentions (e.g. for the session and participant).
(29)	Study team level of "cultural competence"	5	Study team's "objective" ability to provide care to participants with diverse values, beliefs and behaviors, including tailoring delivery to meet participants' social, cultural, and linguistic needs (adapted from Betancourt et al., 2003).
(30)	Study team roles	4	Who does what?
(31)	Facilitators experiential background	4	Personal experience with psychedelics/altered-states of consciousness; personal therapeutic work (y/n)
(32)	Study team commitment to "cultural safety"	3	Related cultural competence, but with a focus on increasing health equity for minority groups. A commitment to "cultural safety" requires the study team to "examine their own positions of privilege and the inherent power imbalances present in therapeutic relationships and encounters" (Lokugamage et al., 2021).
(33)	Facilitator appearance	2	Identifiability and dress (e.g. medical (lab coats), professional, idiosyncratic, shamanic, etc.)

DOSING SESSION(S) (3): Participant experience

#	Item	Frequency	Item details
(34)	Level of physical comfort	36	Participant's level of physical comfort during the dosing session, pertaining to the room set-up and furniture (esp. rest area), the temperature, the air quality, their clothing, their body position, etc.
(35)	Sense of physical safety	21	Sense of physical safety, which may involve: access to medical care; safe interior design; safety from external threats (outsiders, elements); safety from "self" (e.g. locking keys and shoes in a cabinet).
(36)	Sense of cultural congruence	15	Participant's sense of congruence between their identity or culture and the aesthetic / decoration of the room, the sociodemographic characteristics and ideological orientations of people present, and the chosen music, ritual, and language of the session.<br>(*Proxy of the team's level of cultural competence and commitment to cultural safety).
(37)	Level of trust	14	Participant's level of trust in: themselves; in the drug; in the setting; in the people present; in the process/therapy.
(38)	Sense of social safety	11	Sense of social / emotional / psychological safety with the people present, which may involves: clear stopping rules and pre-defined boundaries (e.g. re: touch); confidentiality; (therapeutic) alliance; familiarity.

BROADER PSYCHOTHERAPEUTIC PROCESS (1): Framework

#	Item	Frequency	Item details
(39)	Therapeutic approach	15	Type of psychotherapy provided and manual if possible<br>E.g.: psychoanalytic, ACT, somatic, psychodynamic, EMBARK, integrative PAT approach, loving kindness, etc.
(40)	Drug framing	4	How is the psychedelic drug framed to patients?<br>How are its immediate and lasting effects described?
(41)	Explanatory model	5	General: focus on brain changes (e.g. neuroplasticity), gratitude, mysticism, somating experiencing, new age spirituality, etc.<br>Re illness and recovery: individual vs. collective endeavor; root causes of illness; what it means to be a "good" patient<br>Re the trial: therapeutic, exploratory, promising, scary, etc; being part of a movement (potential pressure to get better)
(42)	Overarching paradigm	4	Biomedical / clinical / neuroscientific vs. spiritual / mystical / religious

BROADER PSYCHOTHERAPEUTIC PROCESS (2): Protocol

#	Item	Frequency	Item details

(43)	Preparation: goals / activities	31	Taking a patient history (incl. prior experience w/ psychedelics)<br>Setting intentions<br>Managing expectations<br>Addressing questions and concerns (e.g., losing control of bodily functions)<br>Psychoeducation (risks vs. benefits; psychedelic effects; how to ask for help during the dosing sessions)<br>Familiarization with the dosing space and people present<br>Family and friends Q&A<br>Independent activities / behaviours (e.g. yoga, meditation, time in nature, disconnecting from the internet)
(44)	Integration: goals / activities	13	Debriefing<br>Interpreting the experience (who does that?)<br>Self-reflection/creative activities<br>Independent activities / behaviours
(45)	Other elements of the protocol	11	Recruitment (incl. Participant information sheet), randomization (incl. protocol for the control group), blinding, consent (incl. consent form), medication wash-out (y/n)
(46)	Number of sessions	6	Number of sessions for preparation, treatment and integration
(47)	Additional support / follow-up	5	Formal: therapeutic (e.g. individual / group therapy), medical (check-ins)<br>Informal: community support, participant advocacy/support groups, other
**MATRIX**			
**#**	**Item**	**Frequency**	**Item details**
(48)	Sociocultural context	14	General: norms/values regarding psychedelics, mental health, emotional processing, transcendent experiences, drugs/ASC<br>Media: Depiction of psychedelics in the media (hype or anti-hype)<br>Legislation: laws and policies surrounding psychedelics<br>Spiritual: overarching religious/spiritual beliefs
(49)	Social determinants of health	12	Social network: family, friends, community and their attitudes toward psychedelics<br>Environmental: home life, housing and major responsibilities<br>Financial: socioeconomic status, employment status and working conditions
	Total codes =	909	Note: 'Frequency' denotes the number of times a code corresponding to a given item appeared in expert free-text responses. One free-text response could generate more than one code, which is why the sum of the 'frequency' column (909) is larger than the number of free-text responses (770) obtained in Round One.

Supplementary Table 3. Round Two subgroup analyses.

Caption: All subgroup analyses conducted following Round Two, including number of experts per subgroup and percentage experts having rated an item as "important" or "very important" per subgroup. Cells highlighted in green indicate items that met the importance (consensus) threshold of 70% for a given subgroup.

Debated Item	Analysis	Subgroup	Count ("Important" + "Very important" responses)	Total # experts in that subgroup	Percentage (count/total)
Bathroom facilities	Whole group	N = 73	44	73	60%
	Gender	Male (n = 38)	24	38	63%
	Gender	Female (n = 31)	19	31	61%
	Gender	Non-binary (n = 4)	1	4	25%
	Ethnicity	White (n = 55)	30	55	55%
	Ethnicity	BIPOC / Mixed (n = 18)	14	18	78%
	Expert Type	Researcher (n = 52)	27	52	52%
	Expert Type	Clinician (n = 31)	17	31	55%
	Expert Type	Participant (n = 20)	16	20	80%
	Field	Psychological (n = 35)	19	35	54%
	Field	Biological (n = 33)	20	33	61%
	Field	Social (n = 26)	15	26	58%
	Field	Plant medicine (n = 4)	1	4	25%
	Clinical Trial Experience	Yes (n = 48)	30	48	63%
	Clinical Trial Experience	No (n = 25)	14	25	56%
Facilitators' level of cultural competence	Whole group	N = 73	38	73	52%
	Gender	Male (n = 38)	22	38	58%
	Gender	Female (n = 31)	14	31	45%
	Gender	Non-binary (n = 4)	2	4	50%
	Ethnicity	White (n = 55)	24	55	44%
	Ethnicity	BIPOC / Mixed (n = 18)	14	18	78%
	Expert Type	Researcher (n = 52)	23	52	44%
	Expert Type	Clinician (n = 31)	14	31	45%
	Expert Type	Participant (n = 20)	13	20	65%
	Field	Psychological (n = 35)	11	35	31%
	Field	Biological (n = 33)	11	33	33%
	Field	Social (n = 26)	17	26	65%
	Field	Plant medicine (n = 4)	4	4	100%
	Clinical Trial Experience	Yes (n = 48)	22	48	46%
	Clinical Trial Experience	No (n = 25)	16	25	64%
Facilitators' commitment to cultural safety	Whole group	N = 73	34	73	47%
	Gender	Male (n = 38)	17	38	45%
	Gender	Female (n = 31)	14	31	45%
	Gender	Non-binary (n = 4)	3	4	75%
	Ethnicity	White (n = 55)	21	55	38%
	Ethnicity	BIPOC / Mixed (n = 18)	13	18	72%
	Expert Type	Researcher (n = 52)	23	52	44%
	Expert Type	Clinician (n = 31)	17	31	55%
	Expert Type	Participant (n = 20)	10	20	50%
	Field	Psychological (n = 35)	11	35	31%
	Field	Biological (n = 33)	11	33	33%
	Field	Social (n = 26)	14	26	54%
	Field	Plant medicine (n = 4)	4	4	100%
	Clinical Trial Experience	Yes (n = 48)	19	48	40%
	Clinical Trial Experience	No (n = 25)	15	25	60%

Odors, scents, smells	Whole group	N = 73	42	73	58%
	Gender	Male (n = 38)	20	38	53%
	Gender	Female (n = 31)	20	31	65%
	Gender	Non-binary (n = 4)	2	4	50%
	Ethnicity	White (n = 55)	30	55	55%
	Ethnicity	BIPOC / Mixed (n = 18)	12	18	67%
	Expert Type	Researcher (n = 52)	25	52	48%
	Expert Type	Clinician (n = 31)	15	31	48%
	Expert Type	Participant (n = 20)	15	20	75%
	Field	Psychological (n = 35)	15	35	43%
	Field	Biological (n = 33)	14	33	42%
	Field	Social (n = 26)	18	26	69%
	Field	Plant medicine (n = 4)	4	4	100%
	Clinical Trial Experience	Yes (n = 48)	26	48	54%
	Clinical Trial Experience	No (n = 25)	16	25	64%
Temperature	Whole group	N = 73	42	73	58%
	Gender	Male (n = 38)	23	38	61%
	Gender	Female (n = 31)	17	31	55%
	Gender	Non-binary (n = 4)	2	4	50%
	Ethnicity	White (n = 55)	27	55	49%
	Ethnicity	BIPOC / Mixed (n = 18)	15	18	83%
	Expert Type	Researcher (n = 52)	26	52	50%
	Expert Type	Clinician (n = 31)	15	31	48%
	Expert Type	Participant (n = 20)	14	20	70%
	Field	Psychological (n = 35)	15	35	43%
	Field	Biological (n = 33)	15	33	45%
	Field	Social (n = 26)	19	26	73%
	Field	Plant medicine (n = 4)	4	4	100%
	Clinical Trial Experience	Yes (n = 48)	28	48	58%
	Clinical Trial Experience	No (n = 25)	14	25	56%
Facilitators demographics and cultural backgrounds	Whole group	N = 73	44	73	60%
	Gender	Male (n = 38)	23	38	61%
	Gender	Female (n = 31)	18	31	58%
	Gender	Non-binary (n = 4)	3	4	75%
	Ethnicity	White (n = 55)	30	55	55%
	Ethnicity	BIPOC / Mixed (n = 18)	14	18	78%
	Expert Type	Researcher (n = 52)	30	52	58%
	Expert Type	Clinician (n = 31)	22	31	71%
	Expert Type	Participant (n = 20)	10	20	50%
	Field	Psychological (n = 35)	23	35	66%
	Field	Biological (n = 33)	17	33	52%
	Field	Social (n = 26)	18	26	69%
	Field	Plant medicine (n = 4)	2	4	50%
	Clinical Trial Experience	Yes (n = 48)	25	48	52%
	Clinical Trial Experience	No (n = 25)	19	25	76%
Sociocultural context	Whole group	N = 73	41	73	56%
	Gender	Male (n = 38)	18	38	47%
	Gender	Female (n = 31)	20	31	65%
	Gender	Non-binary (n = 4)	3	4	75%
	Ethnicity	White (n = 55)	26	55	47%
	Ethnicity	BIPOC / Mixed (n = 18)	15	18	83%
	Expert Type	Researcher (n = 52)	26	52	50%
	Expert Type	Clinician (n = 31)	19	31	61%

	Expert Type	Participant (n = 20)	14	20	70%
	Field	Psychological (n = 35)	15	35	43%
	Field	Biological (n = 33)	15	33	45%
	Field	Social (n = 26)	17	26	65%
	Field	Plant medicine (n = 4)	4	4	100%
	Clinical Trial Experience	Yes (n = 48)	25	48	52%
	Clinical Trial Experience	No (n = 25)	16	25	64%
Social determinants of health	Whole group	N = 73	50	73	68%
	Gender	Male (n = 38)	27	38	71%
	Gender	Female (n = 31)	20	31	65%
	Gender	Non-binary (n = 4)	3	4	75%
	Ethnicity	White (n = 55)	34	55	62%
	Ethnicity	BIPOC / Mixed (n = 18)	16	18	89%
	Expert Type	Researcher (n = 52)	35	52	67%
	Expert Type	Clinician (n = 31)	23	31	74%
	Expert Type	Participant (n = 20)	13	20	65%
	Field	Psychological (n = 35)	22	35	63%
	Field	Biological (n = 33)	20	33	61%
	Field	Social (n = 26)	22	26	85%
	Field	Plant medicine (n = 4)	4	4	100%
	Clinical Trial Experience	Yes (n = 48)	31	48	65%
	Clinical Trial Experience	No (n = 25)	19	25	76%

Supplementary Table 4. Preliminary guidelines presented to experts in Round Four.

Caption: Preliminary guidelines presented to experts in Round Four. The list of 30 items presented therein was final, but the order and wording of items and details was still subject to change based on expert feedback.

Section	Item #	Item details	Location Reported
**DOSING ROOM**			
Location	1	Description of trial location.<br>E.g.: indoors vs. outdoors, place (clinic / hospital / retreat centre / university / home / nature / other) and surroundings (urban / suburban / rural / remote).	
Ambiance	2	Description of ambiance curated by the study team.<br>E.g.: medical / clinical; organic / nature-inspired; ceremonial / shamanic; home-like	
Access to nature	3	Sources of nature or natural elements, if any.<br>E.g.: physical access, windows, in-room flowers / plants, other natural / nature-inspired elements.	
Objects / decorations	4	Description of objects and decorations in the room.<br>E.g.: artwork, "altar", books, candles, photographs, statues, effigies, (wall) colors.<br>Types: religious / spiritual / personally meaningful / grounding / ceremonial / psychedelic.	
Lighting	5	Description of room lighting.<br>E.g., soft / harsh, natural / artificial, bright / dim / dark, tones +/- adjustability.	
Sensory reduction devices	6	Specification of which sensory reduction devices were used, if any.<br>E.g.: eyeshades, earplugs, noise-cancelling headphones	
Toilets	7	Description of:<br>1) Level of accessibility (e.g. near or far from the dosing space)<br>2) Level of privacy (e.g. are interactions with individuals outside of the study team required?)	
**DOSING SESSION: A) Protocol**			
Process / main activities	8	Description of the process and main activities during the dosing session.<br>E.g. inward process (focusing on what emerges internally, e.g. eyes closed +/- listening to music / soundscape) vs. outward process (interacting with others or the environment, e.g. conversation / sharing, chanting, dancing, walking through nature). Note: may be a combination of both.	
Music / other soundscapes	9	Description of the music or soundscape accompanying the trip.<br>E.g.: music vs. (natural) soundscapes vs. voice (e.g. mantras, chants) vs. silence; live vs. recorded; type / genre; playlist; narrative; presence of lyrics; rationale; expected role; continuous vs. intermittent; relation to the trip/process; equipment (e.g., headphones, speakers); sound quality; volume	
Positioning	10	Description of:<br>1) The participant's position (sitting / lying / reclining / walking) and what they are positioned on (bed / sofa / mattress / rug);<br>2) The position of facilitators relative to the participant;<br>3) If group, participant configuration (e.g. circle, line, etc.).	
Dosing schedule	11	If not already reported elsewhere, specification of the dosing schedule.<br>E.g., drug(s) dose, dose frequency, route of administration, continuous vs. intermittent administration, top-up doses; length of the dosing session and breaks; time(s) of dosing (morning, afternoon, evening, night)	
Pre / post-dosing protocol	12	Description of the activities that surrounded dosing.<br>E.g.: spoken / written outline of the day; introduction to objects that could be used during the session (e.g. headphones, weighted blanket); opening / closing ritual and ritual description (e.g., specific use of scents / smells, songs, prayers, etc.); debriefing; food and drink.	
Medical and experimental procedures / assessments	13	Description of medical and experimental procedures and assessments performed, and approximate total duration of testing.<br>1) Medical e.g.: blood draws, PK samples, heart monitoring.<br>2) Experimental e.g.: intensity ratings, psychometric tests, experimental tasks, neuroimaging, interviews.	
Disturbances / interruptions during dosing	14	Reporting of disturbances or interruptions that may have impacted the session, including:<br>1) Lack of privacy: inadequate isolation from the exterior world (e.g. someone unexpectedly entering the space, inadequate sound insulation to ensure confidentiality or a sense of a "container").<br>2) Time or space constraints (rushed process or overcrowded room).<br>3) Sensorial disturbances: auditory (e.g., machines beeping, people talking loudly, traffic sounds, jarring noises, etc.), olfactory (e.g., strong unpleasant smells), visual (e.g. view of hospital wards or ostentive medical equipment), tactile (e.g. inadequate temperature regulation - too hot, cold, humid).	
**DOSING SESSION: B) People Present**			
Who and how many?	15	List of people present during the dosing session, and how many:<br>1) Participant(s): individual vs. group (# in the group)<br>2) Formal support (study team): medical staff, research staff, facilitators<br>3) Informal support: loved ones (friends, family), peers	
Study team skillsets	16	Specification of the study team's qualifications, training (therapeutic / academic / traditional or ancestral practices), history of facilitating / guiding, level of expertise with psychedelics / altered-states of consciousness	
Interpersonal interventions	17	Description of the frequency and type of interpersonal interventions, e.g.:<br>1) Physical (touch, hand-holding, body work)<br>2) Verbal (check-ins, instructions, guidance)	

		3) Non-verbal (supportive silence, aromatherapy)<br>Description of the process for obtaining consent for interpersonal interventions.	
Cultural competency and safety	18	Description of the study team's level of cultural competence (ability to care for participants with diverse social, cultural and linguistic) and commitment to cultural safety.<br>May include: level of diversity within the study team (in terms of languages, cultures and ethnicities represented); recruitment strategies to ensure diversity in the study sample; resources or training programs offered to study staff to gain cultural competency and literacy; anti-oppressive policies.	
Participant autonomy / control / agency	19	Description of the level of control the participant has over:<br>1) Behaviours (e.g., movements like walking or dancing, body position, changing locations);<br>2) Environment (e.g. bringing personal objects, room design/decoration);<br>3) Sensory stimuli (e.g., eye mask on/off, headphones on/off, adjusting lighting or temperature; choice of scents or aromas to accompany their trip);<br>4) Music personalization (on/off, loudness, playlist, genre).	
**BROADER PSYCHOTHERAPEUTIC PROCESS**			
Therapeutic / guiding approach	20	Description of the therapeutic or guiding approach employed during the study.<br>E.g.: structured vs. flexible; standardized vs. individualized; "basic psychological support" vs. specific therapeutic method vs. traditional / ceremonial guiding<br>- If specific therapeutic method: name the method (e.g.: psychoanalytic, ACT, somatic, psychodynamic, EMBARK, integrative PAT approach, loving kindness, etc.) and therapeutic manual if applicable/possible<br>- Other potential specifications: trauma-informed; patient-centered; non-judgmental.	
Narrative framing	21	How is the psychedelic substance framed to the participant?<br>How are its immediate and lasting effects described?<br>- E.g.: mechanistic explanations (e.g. brain changes), gratitude, mysticism, somatic experiencing, new age spirituality.<br>How is the trial described to the participant (by the study team)?<br>- E.g. therapeutic, exploratory, promising; enthusiastically or conservatively.<br>What overarching paradigm is guiding the study team's research question?<br>- E.g. biomedical, neuroscientific, clinical, spiritual, mystical, religious.<br>What are the study team's narratives surrounding illness and recovery?<br>- E.g. individual vs. collective endeavor; root cause of illness.	
Number of sessions	22	Number of and length of sessions for preparation, dosing and integration.	
Preparation protocol	23	Description of the preparation process.<br>E.g.: taking patient history (incl. prior experience w/ psychedelics), setting intentions, managing expectations, addressing questions and concerns (e.g., losing control of bodily functions), psychoeducation (risks vs. benefits, psychedelic effects, how to ask for help during the dosing sessions), familiarization with the dosing space and people present, Q&A for family and friends, independent activities / behaviours (e.g. yoga, meditation, time in nature, disconnecting from the internet)	
Integration protocol	24	Description of the integration process.<br>E.g.: debriefing, interpreting the experience (who does that?), self-reflection / creative activities, independent activities / behaviours.	
Additional support / follow-up	25	Description of the additional support and follow-up offered to participants after the end of the trial.<br>1) Formal e.g.: therapeutic support (e.g. individual / group therapy), medical support (check-ins, additional monitoring / medications for individuals with adverse / destabilizing effects); expectation management (re: future access to psychedelic treatments, given current legislation).<br>2) Informal e.g.: community support, peer support, participant advocacy/support groups, family and friends support (including re: ppt's involvement in this clinical trial).	
**PARTICIPANT EXPERIENCE**			
Therapeutic alliance	26	Measurement of therapeutic alliance between participant and each facilitator.	
Level of trust	27	Measurement of the participant's level of trust during the dosing sessions, including trust in: themselves (to be able to "let go"); in the drug; in the setting; in the people present; in the process/therapy.	
Level of physical comfort	28	Measurement of the participant's level of physical comfort during the dosing session.<br>May pertain to: the room set-up and furniture (esp. the dosing bed / chair / mat), the temperature, the air quality, their clothing, their body position, toilet accessibility.	
Sense of physical safety	29	Measurement of the participant's sense of physical safety during the dosing session.<br>May pertain to: access to medical care; safe interior design; safety from external threats.	
Sense of social and cultural safety	30	Measurement of the participant's sense of social / psychological and cultural safety with the people present.<br>May pertain to: boundaries (physical, psychological) being respected; familiarity with people present; sense that cultural background is respected, accommodated and understood.	

Supplementary Table 5. Progression of items from Round 1 to Round 3, and corresponding item name in final guidelines.

Caption: Progression of each item between Round One and Round Three, including which items reached consensus in the whole group (green), which items were subject to debate (orange) for reaching consensus only in certain subgroups, and which items were rejected (red). Also indicates which items required adjustments (yellow), and how they were adjusted.

**Round One**		**Round Two**			**Round Three**	**Round Four**	
**Item #**	**Original item**	**Consensus in the whole group?** (consensus score)	**Consensus in at least one subgroup?**	**Acceptable as is?** If N: what must be done	**Final status** (final whole group consensus score) **denotes a change in score	**Final item**	**Item #**
1	Objects / decorations / artworks	Y (70%)	N/a	Y	Approved (70%)	Objects and decoration	4
2	Nature	Y (71%)	N/a	Y	Approved (71%)	Access to nature	3
3	Facilities	N (60%)	Y: Debate	N: Modify	Approved (73%**)	Bathroom facilities	7
4	Location	Y (86%)	N/a	Y	Approved (86%)	Location	1
5	Rest area	Y (74%)	N/a	N: Modify	Approved (74%)	Positioning	9
6	Layout / positioning	N (59%)	Y: Debate	N: Modify + Combine (5)	Combined to item 5		
7	Equipment	N (53%)	N: Rejected				
8	Lighting	Y (77%)	N/a	Y	Approved (77%)	Lighting	5
9	Global "feel"	Y (84%)	N/a	N: Modify	Approved (84%)	Ambiance	2
10	Sensory reduction devices	Y (80%)	N/a	Y	Approved (80%)	Sensory reduction	6
11	Acoustics	Y (76%)	N/a	N: Combine (18)	Combined to item 18		
12	Odors / scents / smells	N (58%)	Y: Debate	Y	Rejected (64%**)		
13	Temperature	N (57%)	Y: Debate	Y	Rejected (63%**)		
14	Other	N (26%)	N: Rejected				
15	Music / other soundscapes	Y (92%)	N/a	Y	Approved (92%)	Music or soundscapes	11
16	Participant autonomy / control / agency	Y (93%)	N/a	Y	Approved (93%)	Participant autonomy, control, and agency	13
16	Supportive interventions	Y (96%)	N/a	Y	Approved (96%)	Interpersonal interventions	12
18	Disturbances / interruptions	Y (83%)	N/a	N: Combine (11)	Approved (83%) - combination accepted	Potential disturbances or interruptions	17
19	Approach	Y (87%)	N/a	N: Modify + Combine (23)	Combined to item 25 instead of 23		
20	Procedures	Y (79%)	N/a	Y	Approved (79%)	Experimental and medical procedures or assessments	15
21	Pre / post-dosing protocol	Y (85%)	N/a	Y	Approved (85%)	Pre- and post-dosing protocol	16
22	Dosing schedule	Y (80%)	N/a	N: Modify	Approved (80%)	Dosing regimen	14
23	Process / main activities	Y (78%)	N/a	N: Modify + Combine (19)	Approved (78%) - combination rejected	Focus and main activities	10
24	Who and how many?	Y (95%)	N/a	Y	Approved (95%)	Number and roles of people present	8
25	(Therapeutic) alliance	Y (89%)	N/a	N: Reconsider	Approved (89%) - move to "Participant Experience"	Therapeutic alliance	26
26	Study team professional backgrounds and skillsets	Y (79%)	N/a	Y	Approved (79%)	Study personnel qualifications	23
27	Facilitators and other participant(s) demographics / cultural backgrounds	N (61%)	Y: Debate	N: Modify	Rejected (53%**)		
28	Study team mindsets	N (61%)	Y: Debate	N: coherence too low – rejected			
29	Study team level of "cultural competence"	N (52%)	Y: Debate	N: Modify + Combine	Combined to item 32		
30	Study team roles	N (43%)	N: Rejected	n/a			
31	Facilitators experiential background	N (55%)	Y: Debate	N: privacy issues – rejected			

32	Study team commitment to "cultural safety"	N (49%)	Y: Debate	N: Modify + Combine	Approved (73%**) - combination accepted	Cultural competence and safety	25
33	Facilitator appearance	N (29%)	N: Rejected				
34	Level of physical comfort	Y (79%)	N/a	N: Reconsider	Approved (79%) - move to "Participant Experience"	Physical comfort	28
35	Sense of physical safety	Y (81%)	N/a	N: Reconsider	Approved (81%) - move to "Participant Experience"	Physical safety	29
36	Sense of cultural congruence	N (54%)	Y: Debate	N: Modify + Combine	Combined to item 38		
37	Level of trust	Y (85%)	N/a	N: Reconsider	Approved (85%) - move to "Participant Experience"	Trust	27
38	Sense of social safety	Y (86%)	N/a	N: Modify + Reconsider	Approved (86%) - move to "Participant Experience"	Psychological and cultural safety	30
39	Therapeutic approach	Y (85%)	N/a	Y	Approved (85%)	Therapeutic or guiding approach	18
40	Drug framing	Y (80%)	N/a	N: Modify + Combine (41, 42)	Combined with item 41 and 42		
41	Explanatory model	Y (73%)	N/a	N: Modify + Combine (40, 42)	Combined with item 40 and 42		
42	Overarching paradigm	Y (75%)	N/a	N: Modify + Combine (40, 41)	Approved (75%) - mod. + combo accepted	Narrative framing	19
43	Preparation: goals / activities	Y (98%)	N/a	Y	Approved (98%)	Preparation protocol	21
44	Integration: goals / activities	Y (94%)	N/a	Y	Approved (94%)	Integration protocol	22
45	Other elements of the protocol	Y (80%)	N/a	N: standard reporting			
46	Number of sessions	Y (85%)	N/a	Y	Approved (85%)	Number and length of sessions	20
47	Additional support / follow-up	Y (92%)	N/a	Y	Approved (92%)	Additional support or follow-up	23
48	Sociocultural context	N (57%)	Y: Debate	N: Modify	Rejected (44%**)		
49	Social determinants of health	N (69%)	Y: Debate	N: Modify	Rejected (64%**)		

Supplementary Table 6. Round Four results.

Caption: Results from the fourth round of the Delphi study, including expert responses on perceived quality of the Preliminary guidelines, the Delphi process, their personal experience of the Delphi and their conception of setting.

Prompts	Rating	Count	Percentage
**The preliminary guidelines**			
Overall satisfaction with the guidelines	Very satisfied	31	50%
N = 62	Satisfied	28	45%
	Neutral	2	3%
	Unsatisfied	1	2%
	Very unsatisfied	0	0%
They reflect what is important "to me".	Strongly agree	16	26%
N = 62	Agree	43	69%
	Neutral	2	3%
	Disagree	1	2%
	Strongly disagree	0	0%
They reflect what is important to report (in clinical trials with psychedelics).	Strongly agree	22	35%
N = 62	Agree	37	60%
	Neutral	2	3%
	Disagree	1	2%
	Strongly disagree	0	0%
I would use these guidelines myself.*	Yes, definitely.	43	74%
N = 58	Maybe, I would consider it	14	24%
	No, I would not use them	1	2%
**The Delphi process**			
Clarity of the instructions	Extremely clear	26	46%
N = 57	Mostly clear	29	51%
	Moderately clear	2	4%
	Unclear	0	0%
	Extremely unclear	0	0%
Satisfaction with structure and organization	Very satisfied	35	61%
N = 57	Satisfied	19	33%
	Neutral	3	5%
	Unsatisfied	0	0%
	Very unsatisfied	0	0%
The deadlines were reasonable.	Strongly agree	27	47%
N = 57	Agree	29	51%
	Neutral	1	2%
	Disagree	0	0%
	Strongly disagree	0	0%
The time commitment was reasonable.	Strongly agree	27	47%
N = 57	Agree	27	47%
	Neutral	2	4%
	Disagree	1	2%
	Strongly disagree	0	0%
Any technical difficulties?	Yes	5	9%
N = 57	No	52	91%
**Personal experience**			
My expertise and opinions were adequately considered	Strongly Agree	26	46%
N = 57	Agree	28	49%
	Neutral	1	2%
	Disagree	1	2%

	Strongly disagree	0	0%
I've learned and expanded my knowledge throughout this Delphi process.	Strongly Agree	20	35%
N = 57	Agree	23	40%
	Neutral	14	25%
	Disagree	0	0%
	Strongly disagree	0	0%
I felt comfortable expressing my opinion during the Delphi process.	Strongly Agree	35	61%
N = 57	Agree	19	33%
	Neutral	2	4%
	Disagree	1	2%
	Strongly disagree	0	0%
This Delphi process successfully identified areas of agreement and disagreement among experts.	Strongly Agree	23	40%
N = 57	Agree	33	58%
	Neutral	0	0%
	Disagree	1	2%
	Strongly disagree	0	0%
First time taking part in a Delphi	Yes	48	84%
N = 57	No	9	16%
Would take part again	Yes	57	100%
N = 57	No	0	0%
**Conception of setting**			
My conception of "setting" has changed (since the start of the Delphi)	Yes, significantly	2	4%
N = 57	Yes, somewhat	37	65%
	No, not at all	18	32%
Set and "setting" can be studied separately.	Strongly Agree	2	4%
N = 57	Agree	19	33%
	Neutral	12	21%
	Disagree	21	37%
	Strongly disagree	3	5%
Where does "setting" begin and end?	From the start to the end of the dosing session	5	9%
N = 57	From the start of preparation to the end of integration	8	14%
	From the start of recruitment to the end of follow-up	40	70%
	Other: “Impossible to say”; “Forever”; “Depends on the participant”; “I think we can differentiate the setting for each of the above - so we can talk about the setting on the dosing day, the setting of the trial etc.”	4	7%

Supplementary Table 7. Concerns raised by experts regarding the preliminary guidelines.

Experts' Concerns	Actions Taken by Study Leads
Excessive length or level of detail of the preliminary guidelines.	The final ReSPCT guideline was simplified, with additional details moved to the explanatory document for clarity.
Research teams may not be able to address all the items in the main body of their manuscripts due to word count restrictions.	Clarified that the 30 items can be reported either in the main articles or the supplements of future studies (see Table 3 caption).
Certain items may have little chance of reaching statistical significance despite data pooling across studies – either due to being too specific or hard to measure quantitatively.	Acknowledged that the guidelines represent a preliminary consensus and that further empirical research is required to evaluate the actual importance of the variables therein (Limitations section).
Certain items will not be relevant to certain study contexts, and thus the guidelines may need to be adapted by study teams.	Acknowledged that the guidelines may be adapted depending on study context (Discussion section).

Supplementary Table 8. Completed Pre-Recommendations for the Conducting and REporting of DElphi Studies (CREDES) Checklist

Caption: Table derived frrom Junger S, Payne SA, Brine J, Radbruch L, Brearley SG. Guidance on Conducting and REporting DElphi Studies (CREDES) in palliative care: Recommendations based on a methodological systematic review. Palliative Medicine 2017;31:684-706.

Section/Topic	#	Item description	Location reported
**Rationale for the choice of the Delphi technique**			
Justification.	1	The choice of the Delphi technique as a method of systematically collating expert consultation and building consensus needs to be well justified. When selecting the method to answer a particular research question, it is important to keep in mind its constructivist nature.	Introduction
**Planning and design**			
Planning and process.	2	The Delphi technique is a flexible method and can be adjusted to the respective research aims and purposes. Any modifications should be justified by a rationale and be applied systematically and rigorously.	Methods and Results (Delphi Rounds)
Definition of consensus.	3	Unless not reasonable due to the explorative nature of the study, an a priori criterion for consensus should be defined. This includes a clear and transparent guide for action on (a) how to proceed with certain items or topics in the next survey round, (b) the required threshold to terminate the Delphi process and (c) procedures to be followed when consensus is (not) reached after one or more iterations.	Methods
**Study conduct**			
Informational input.	4	All material provided to the expert panel at the outset of the project and throughout the Delphi process should be carefully reviewed and piloted in advance in order to examine the effect on experts' judgements and to prevent bias	Results (Delphi Rounds)
Prevention of bias.	5	Researchers need to take measures to avoid directly or indirectly influencing the experts' judgements. If one or more members of the research team have a conflict of interest, entrusting an independent researcher with the main coordination of the Delphi study is advisable	N/A; no conflicts of interest among the study leads.
Interpretation and processing of results.	6	Consensus does not necessarily imply the 'correct' answer or judgement; (non)consensus and stable disagreement provide informative insights and highlight differences in perspectives concerning the topic in question.	Results
External validation.	7	It is recommended to have the final draft of the resulting guidance on best practice in palliative care reviewed and approved by an external board or authority before publication and dissemination	N/A
**Reporting**			
Purpose and rationale.	8	The purpose of the study should be clearly defined and demonstrate the appropriateness of the use of the Delphi technique as a method to achieve the research aim. A rationale for the choice of the Delphi technique as the most suitable method needs to be provided.	Introduction
Expert panel.	9	Criteria for the selection of experts and transparent information on recruitment of the expert panel, socio- demographic details including information on expertise regarding the topic in question, (non)response and response rates over the ongoing iterations should be reported.	Methods (Expert Panel)
Description of the methods.	10	The methods employed need to be comprehensible; this includes information on preparatory steps (How was available evidence on the topic in question synthesised?), piloting of material and survey instruments, design of the survey instrument(s), the number and design of survey rounds, methods of data analysis, processing and synthesis of experts' responses to inform the subsequent survey round and methodological decisions taken by the research team throughout the process.	Methods
Procedure.	11	Flow chart to illustrate the stages of the Delphi process, including a preparatory phase, the actual 'Delphi rounds', interim steps of data processing and analysis, and concluding steps.	Figure 1
Definition and attainment of consensus.	12	It needs to be comprehensible to the reader how consensus was achieved throughout the process, including strategies to deal with non-consensus.	Methods and Results
Results.	13	Reporting of results for each round separately is highly advisable in order to make the evolving of consensus over the rounds transparent. This includes figures showing the average group response, changes between rounds, as well as any modifications of the survey instrument such as deletion, addition or modification of survey items based on previous rounds.	Results
Discussion of limitations.	14	Reporting should include a critical reflection of potential limitations and their impact of the resulting guidance.	Discussion (Limitations)
Adequacy of conclusions.	15	The conclusions should adequately reflect the outcomes of the Delphi study with a view to the scope and applicability of the resulting practice guidance.	Results (Round Four)
Publication and dissemination.	16	The resulting guidance on good practice in palliative care should be clearly identifiable from the publication, including recommendations for transfer into practice and implementation. If the publication does not allow for a detailed presentation of either the resulting practice guidance or the methodological features of the applied Delphi technique, or both, reference to a more detailed presentation elsewhere should be made (e.g. availability of the full guideline from the authors or online; publication of a separate paper reporting on methodological details and particularities of the process (e.g. persistent disagreement and controversy on certain issues)). A dissemination plan should include endorsement of the guidance by professional associations and health care authorities to facilitate implementation.	Supplement (Explanatory Document)

Supplement 1

ReSPCT Guidelines 2025 – Reporting Table

Pronovost-Morgan C, Greenway KT, Roseman L, The ReSPCT Experts. An international Delphi consensus for reporting of setting in psychedelic clinical trials. *Nat Med.* 2025. https://doi.org/10.1038/s41591-025-03685-9

Item	Item Number	Item details	Reported on page number:
**Physical environment**			
Location	1	Location of the trial, and whether indoors/outdoors and urban/rural/suburban.	
sAmbiance	2	Room ambiance curated by the study team.	
Access to nature	3	Sources of nature or natural elements that are physically or visually accessible to participants.	
Objects and decorations	4	Objects and decorations in the room.	
Lighting	5	Room lighting and adjustability.	
Sensory reduction	6	Sensory reduction used, such as headphones and eyeshades.	
Bathroom facilities	7	Level of bathroom accessibility and privacy.	
**Dosing session procedure**			
Number and roles of people present	8	Number and roles of people present, including participants, study staff and informal support.	
Positioning	9	Relative position of people in the room, and what participants were positioned on (e.g., bed, mat, couch).	
Focus and main activities	10	Focus (internal or external) and main activities of the dosing session.	
Music or soundscapes	11	Music or soundscapes that accompanied the dosing experience.	
Interpersonal interventions	12	Verbal or physical interpersonal interventions used throughout the session, and how consent was obtained.	
Participant autonomy, control, and agency	13	Level of participant control and agency over activities and environment of the dosing session.	
Dosing regimen	14	Dosing regimen, including drug dose(s), frequency, route of administration and length of the dosing session.	
Medical and experimental procedures or assessments	15	Medical and experimental procedures or assessments performed during the dosing session.	
Pre- and post-dosing protocol	16	Activities that took place immediately prior to- or post-dosing, including participant arrival and release conditions.	
Potential disturbances or interruptions	17	Disturbances or interruptions that may have impacted the quality of the dosing session.	
**Therapeutic framework and protocol**			
Therapeutic or guiding approach	18	Therapeutic/guiding approach used throughout the study, if any, with the accompanying manual or protocol.	
Narrative framing	19	Framing of the trial intervention by the study team, including the short- and long-term drug effects.	
Number of sessions	20	Number and length of preparation, dosing, and integration sessions.	

Preparation protocol	21	Activities performed during the preparation sessions.	
Integration protocol	22	Activities performed during the integration sessions.	
Additional support/follow-up	23	Formal or informal support or follow-up offered to participants after the end of the trial.	
Study personnel qualifications	24	The credentials, training, and expertise of personnel providing the study intervention or care.	
Cultural competence and safety	25	Study team's level of cultural competence and efforts towards cultural safety.	
**Subjective experiences**			
Therapeutic alliance	26	Therapeutic alliance between participants and facilitators throughout the intervention.	
Trust	27	Participant's level of trust throughout the intervention.	
Physical comfort	28	Participant's level of physical comfort during the dosing session.	
Physical safety	29	Participant's sense of physical safety during the dosing session.	
Psychological and cultural safety	30	Participant's sense of interpersonal safety with the people present during the session.	

Supplement 2

ReSPCT Guidelines 2025 – Explanatory Document

An international Delphi consensus for reporting of Setting in Psychedelic Clinical Trials

Pronovost-Morgan, C., Greenway, K.T., Roseman, L., & ReSPCT Experts:

[Aday, J.S., Agin-Liebes, G., Ahmad, K., Aicher, H., Aixalà, M., Amar, S., Araujo, D., Barrett, F.S., Belser, A., Blainey, M., Bowman, R., Bourne, M., Breeksema, J., Breelove Carter, K., Bussey, J., Camara, C., Canby, N., Chalchi, J., Close, J., Cox, T., Daraeizadeh, P., Davis, A., de la Salle, S., Devenot, N., Dyck, E., Erritzoe, D., Farzin, H., Friesen, P., Garel, N., Giblin, J., Golden, T., Greer, G., Gukasyan, N., Guss, J., Hapke, E., Hartogsohn, I., Holze, F., Kettner, H., Knowles, A., Langlitz, N., Lehmann, A., Lifshitz, M., Lucas, P., MacLean, K., Mallon, O., Marcus, O., Mazarrasa, J., McAlpine, R., Moreno, F., Neitzke-Spruill, L., Noorani, T., Oehen, P., Ona, G., Palhano-Fontes, F., Patchett-Marble, R., Peekeekoot, G., Perkins, D., Preller, K.H., Ramaekers, J.G., Reed, S., Repantis, D., Richards, B., Richards, W., Risby, S., Rochester, J., Roullier, I., Rucker, J., Ruffell, S., Sanders, J.W., Schneider, L., Shutte, T., Szigeti, B., Tadwalkar, S., Tzarfaty, K., Thibault Lévesque, J., Timmermann, C., Wells, H., Williams, K.]

Introduction

The Reporting of Setting in Psychedelic Clinical Trials (ReSPCT) Guidelines 2025 were produced by an international Delphi consensus study regarding the most important extra-pharmacological factors in psychedelic clinical trials. Extra-pharmacological factors refer to the contexts that a psychedelic drug is administered in, commonly described as “(mind)set and setting” (Hartogsohn, 2017). These guidelines are designed to facilitate the standardized reporting of such contextual variables in clinical trials and to generally foster rigorous research on the influences of contexts on psychedelic drug effects.

The following explanatory document is complementary to the published guidelines, which are reproduced below and available as a fillable document as an appendix to the main publication. This document is intended as a guide for clinicians and researchers to better understand and report the most important contextual influences on psychedelic drugs as per the consensus established by the ReSPCT Delphi study.

Each item should be reported in publications of clinical trials involving psychedelic drugs, either in the main article or as supplementary information, towards increasing the validity and transparency of this growing body of literature. In the case of multisite studies, it is recommended to report differences in individual sites regarding these items (e.g. through a photograph of the unique dosing locations).

The first three categories of the guidelines pertain to essential elements of the trial that may be understood as the ‘setting’ variables. They should be directly recorded and reported by the study team.

The last category pertains to key elements of the participant experience, which may be described as ‘(mind)set’ variables. These items are, by definition, more subjective, and individual-dependent than the items of the first three sections, and thus require more consideration for their evaluation. They can be assessed via standardized questionnaires, potentially at multiple timepoints, and/or by participant interviews. Examples of existing standardized measures are provided for each item in this section. Given that these items place additional burdens on study teams and participants, they are not considered obligatory for all psychedelic clinical trials but warrant consideration.

In this document, each item is accompanied by an explanatory paragraph with relevant citations where available, suggested reporting prompts, and representative quotes from the Delphi study experts that motivated the item’s inclusion in the reporting guidelines.

Although designed specifically for psychedelic clinical trials, these contextual items may warrant consideration and reporting in other forms of research, including clinical trials involving other psychoactive substances and observational or cross-sectional studies of psychedelic drugs.

The ReSPCT 2025 Guidelines

Item	Item Number	Item details
**Physical environment**		
Location	1	Location of the trial, and whether indoors/outdoors and urban/rural/suburban.
Ambiance	2	Room ambiance curated by the study team.
Access to nature	3	Sources of nature or natural elements that are physically or visually accessible to participants.
Objects and decorations	4	Objects and decorations in the room.
Lighting	5	Room lighting and adjustability.
Sensory reduction	6	Sensory reduction used, such as headphones and eyeshades.
Bathroom facilities	7	Level of bathroom accessibility and privacy.
**Dosing session procedure**		
Number and roles of people present	8	Number and roles of people present, including participants, study staff and informal support.
Positioning	9	Relative position of people in the room, and what participants were positioned on (e.g., bed, mat, couch).
Focus and main activities	10	Focus (internal or external) and main activities of the dosing session.
Music or soundscapes	11	Music or soundscapes that accompanied the dosing experience.
Interpersonal interventions	12	Verbal or physical interpersonal interventions used throughout the session, and how consent was obtained.
Participant autonomy, control, and agency	13	Level of participant control and agency over activities and environment of the dosing session.
Dosing regimen	14	Dosing regimen, including drug dose(s), frequency, route of administration and length of the dosing session.
Medical and experimental procedures/assessments	15	Medical and experimental procedures or assessments performed during the dosing session.
Pre- and post-dosing protocol	16	Activities that took place immediately prior to- or post-dosing, including participant arrival and release conditions.
Potential disturbances or interruptions	17	Disturbances or interruptions that may have impacted the quality of the dosing session.
**Therapeutic framework and protocol**		
Therapeutic/guiding approach	18	Therapeutic/guiding approach used throughout the study, if any, with the accompanying manual or protocol.
Narrative framing	19	Framing of the trial intervention by the study team, including the short- and long-term drug effects.
Number of sessions	20	Number and length of preparation, dosing, and integration sessions.
Preparation protocol	21	Activities performed during the preparation sessions.
Integration protocol	22	Activities performed during the integration sessions.
Additional support/follow-up	23	Formal or informal support or follow-up offered to participants after the end of the trial.
Study personnel qualifications	24	The credentials, training, and expertise of personnel providing the study intervention or care.
Cultural competence and safety	25	Study team's level of cultural competence and efforts towards cultural safety.
**Subjective experiences**		
Therapeutic alliance	26	Therapeutic alliance between participants and facilitators throughout the intervention.
Trust	27	Participant's level of trust throughout the intervention.
Physical comfort	28	Participant's level of physical comfort during the dosing session.
Physical safety	29	Participant's sense of physical safety during the dosing session.
Psychological and cultural safety	30	Participant's sense of interpersonal safety with the people present during the session.

Explanatory Document: Table of Contents

Dosing environment ***22***

Item 1. Location 22

Item 2. Ambiance 23

Item 3. Access to nature 24

Item 4. Objects and decorations 25

Item 5. Lighting 26

Item 6. Sensory reduction 27

Item 7. Bathroom facilities 28

Dosing session procedure ***29***

Item 8. Number and role of people present 29

Item 9. Positioning 30

Item 10. Focus and main activities 31

Item 11. Music or soundscapes 32

Item 12. Interpersonal interventions 33

Item 13. Participant autonomy, control, and agency 34

Item 14. Dosing regimen 35

Item 15. Medical and experimental procedures or assessments 36

Item 16. Pre- and post-dosing protocol 37

Item 17. Potential disturbances or interruptions 38

Therapeutic framework and protocol ***39***

Item 18. Therapeutic or guiding approach 39

Item 19. Narrative framing 40

Item 20. Number of sessions 41

Item 21. Preparation protocol 42

Item 22. Integration protocol 43

Item 23. Additional support or follow-up 44

Item 24. Study personnel qualifications 45

Item 25. Cultural competence and safety 46

Subjective evaluation of the setting ***47***

Item 26. Therapeutic alliance 47

Item 27. Trust 48

Item 28. Physical comfort 49

Item 29. Physical safety 50

Item 30. Psychological and cultural safety 51

Dosing environment

Item 1. Location

Specific location of the trial, and whether indoors/outdoors and urban/rural/suburban.

Drug experiences are known to be influenced by the physical environment in which they take place. Whereas many traditional, underground and recreational usages of psychedelics involve outdoors and nature-based settings (Lawlor, 2013), clinical trials typically occur indoors due to the control, safety, predictability, and privacy offered by such environments (Cooley et al., 2020). Beyond being indoors or outdoor, the specific site location (e.g., a hospital room vs. a retreat center) and its greater surroundings (urban vs. rural) may have psychological effects on participants before, during, and after their psychedelic experience(s) (Studerus et al., 2012).

<u>Suggested reporting questions</u>:

- Did dosing sessions take place indoors or outdoors?
- Where did the intervention take place? For instance, in a private clinic, a hospital, a retreat center, an academic institution, a private residence, or in a natural setting.
- What were the broader surroundings? For instance, urban, suburban, rural, or remote.

<u>Selected relevant quotes of Delphi consensus process experts</u>:

- “Outside trip room environment - e.g., is it conducted in a psychedelic clinic or a hospital or someone's living room?”
- “A hospital/medical setting might instill greater trust and comfort in some, while for others it could influence the emotional or visual nature of their experience in different ways.”
- “Regarding surroundings: "[...] compare the crime-ridden streets around Johns Hopkins with passing by sheep on green pastures on the way to the Psychiatric Hospital in Zurich.”

Item 2. Ambiance

Room ambiance curated by the study team.

In addition to the physical location of a trial, the study team may intend to curate a specific ambiance or atmosphere for the dosing space. For instance, a hospital setting may be considered highly medicalized or anxiogenic (Eisner, 1997), but be adapted to instill a sense of warmth or peace. Indeed, psychedelic-assisted therapy trials often occur in rooms within medical or academic institutions that have been setup to create 'living-room like' atmospheres (Johnson et al., 2008; Noorani, 2021). Conversely, trials that involve significant physiological measurements or neuroimaging may necessarily convey 'clinical' or 'medical' ambiances (Studerus et al., 2012), and trials investigating traditional indigenous settings may foster 'ceremonial' or 'shamanic' ambiances (Loizaga-Velder & Verres, 2014; Sapoznikow et al., 2019).

Suggested reporting questions:

- Was a particular ambiance curated by the study team? For instance, medical/clinical or purposefully de-medicalized?
- Was there a particular guiding aesthetic to this ambiance? For instance, nature-inspired, psychedelia-inspired, ceremonial/shamanic, or living room/home-like?

Selected relevant quotes of Delphi consensus process experts:

- "The intention the study team had in setting up the room - what overall ambience did they want to achieve?"
- "Medical vs. home vs. shamanic setting; if medical setting, does the room look like a hospital room?"
- "Feel of the room - Needs to have a calm aura and feel welcoming."

Item 3. Access to nature

Sources of nature or natural elements that are physically or visually accessible to participants.

Psychedelic effects are thought to have particularly strong interactions with nature, in ways that have potential impacts on psychedelic experiences and on health-related outcomes, including psychedelic-induced increases in 'nature-relatedness' (Kettner et al., 2019). Nature-based settings are also known to have psychologically soothing properties in-and-of themselves, which may be amplified by psychedelic drug effects and/or complement the effectiveness of psychedelic therapies (Gandy et al., 2020). In Indigenous contexts, nature is often at the center of psychedelic ritual (Williams & Brant, 2023). Although clinical trials are less often conducted outdoors, indoor research settings may offer visual or physical access to nature, and/or be decorated with reminders of the natural world.

Suggested reporting questions:

- Were any sources of nature present during dosing sessions? If so, what types?
    - Visual access to nature, for instance, through a window.
    - Natural elements, like plants or flowers.
    - Other reminders of nature, like nature-based art and photography.
    - Biophilic design elements designed to mimic the feeling of being outdoors.
- Did participants have physical access to nature? For instance, did treatment or other sessions take place outdoors or were participants able to go outside during the session?

Selected relevant quotes of Delphi consensus process experts:

- "Access to nature or view of nature - Given typically increased nature-relatedness, this is an important factor."
- "From my way of understanding, natural elements have a spirit, as important as humans in the psychedelic setting."
- "Presence of nature - Experiences with nature are often highly meaningful for people who take part in experiences or ceremonies within or adjacent to natural scenery. Although the majority of scientific trials take place indoors, many treatment sites attempt to approximate this effect by including plants or decorations that mimic natural scenery."

Item 4. Objects and decorations

Objects and decorations in the room.

The ambiance curated by the study team (Item 2) partly relies on the objects and decorations included in the dosing environment (Langlitz, 2013). Item 4 allows the study team to detail such objects, to convey a sense of the physical dosing space and to describe the main visual stimuli exposed to participants during dosing sessions. Such objects may influence the nature of the psychedelic experience itself (Johnson, 2020), with such effects dependent on participants' sociocultural backgrounds and previous life experiences (Williams et al., 2020). Item 4 may be reported in written form, and/or with photographs of the dosing environment.

Suggested reporting questions:

- What were the salient objects and decorations in the dosing space?
    - For instance, artwork, altar/center, books, candles, photographs, statues, effigies, ritual objects, or blankets.
- What were the desired connotations and/or purposes of these objects?
    - For instance, religious, spiritual, psychedelic, ceremonial, grounding, or personally/culturally meaningful.
- Were participants given the opportunity to bring familiar objects?
- Was cultural diversity considered in the choice of objects and decorations?

Selected relevant quotes of Delphi consensus process experts:

- "What is the predominant aesthetic and are there objects with connotations in the setting (spiritual; substance-related such as mushrooms etc.); corporate identifying objects."
- "Personalized or impersonalized space - participant's own home versus a novel space, opportunity to bring familiar objects or surrounded by unfamiliar stimuli."
- "Options for the use of ritual objects - The importance of this factor really depends on the participants. If your group is mostly Indigenous, or has animist leanings, having ritual objects such as feathers, shells for smudging, and more could be very important."

Item 5. Lighting

Room lighting and adjustability.

Lighting is known to impact mood (Kong et al., 2022) and research has found that subjects under the influence of psilocybin report specific lighting preferences, such as for dimmer light at peak drug effects (Fischer et al., 1969). Furthermore, psychedelic users report the level of lighting can influence the emotional content of psychedelic experiences (Palmer & Maynard, 2022), potentially enhancing or supporting psychedelic experiences.

Suggested reporting questions:

- Was the lighting bright, dim, or dark during dosing session?
- Was the lighting natural or artificial?
- Was lighting adjustable? If so, by what means? For instance, by dimmers, lamps, blinds, or curtains?

Selected relevant quotes of Delphi consensus process experts:

- "I think it's important to be able to control the artificial lighting (e.g., dimmer switch) and external natural light (e.g., block out blinds)."
- "Lighting can generally have a profound effect on mood and emotional regulation, as well as the aesthetic quality of a space. Poor lighting could derail a psychedelic experience."
- "All psychedelics I have worked with provide different visions depending on the light."

Item 6. Sensory reduction

Sensory reduction used, such as headphones and eyeshades.

Blindfolds, headphones, and other tools that reduce external sensorial input have been shown to influence the phenomenology of drug experiences (Swanson, 2018). For instance, closed-eye and open-eye perceptual changes induced by serotonergic psychedelics (Kometer & Vollenweider, 2018) or ketamine (Garel et al., 2023) can significantly differ. Similarly, the use of immersive audio-listening equipment such as headphones are thought to lead to more 'inner experiences' by reducing the impacts of external sounds as well as interactions between participants and practitioners (Garcia-Romeu & Richards, 2018). Many, but not all, psilocybin-assisted psychotherapy trials provide blindfolds and headphones to participants, and encourage them to employ them for most of the treatment session (Johnson et al., 2008). Protocols relating to sensory reduction will be crucial to specify given their potential impact on the psychedelic experience (Langlitz, 2013; Oehen & Gasser, 2022).

Suggested reporting questions:

- Were blindfolds provided? If so, what variety?
- Were headphones provided? If so, what variety?
- Were participants actively encouraged to make use of blindfolds and/or headphones throughout dossing sessions? Were there any restrictions as to how long they could go without them?

Selected relevant quotes of Delphi consensus process experts:

- "Whether an eyeshade is employed, its type/quality, and rules around adherence to eyeshades (e.g., whether participants are strongly encouraged to keep eyeshades on) can be significant to participant experiences."
- "Sensory isolation, such as the use of eye masks or earplugs, can help participants focus on their inner experiences and reduce external distractions, leading to potentially deeper and more transformative experiences."
- "I think some people might find the goggles and headphones to feel quite claustrophobic."

Item 7. Bathroom facilities

Level of bathroom accessibility, privacy, and safety.

Psychedelic substances can induce feelings of disorientation and confusion in unfamiliar environments or with unfamiliar people. In psychedelic studies, this may occur when participants only have access to bathroom facilities that are removed from the insular dosing space. Researchers from the Johns Hopkins Center for Psychedelic and Consciousness Research reported such an incident, whereby a volunteer felt confused while in the bathroom during a dosing session (Carbonaro et al., 2016). They since adjusted and detailed their protocol to ensure bathroom accessibility and safety, providing an example of how this may be reported in the literature.

Suggested reporting questions:

- How close or far were the bathroom facilities in relation to the dosing space?
- How accessible were the bathroom facilities?
- How private were the bathroom facilities? Did participants have to interact with the "outside world" and/or individuals outside of the study team on their way to or from the bathroom?
- Were there any measures in place to ensure the safety of participants while they used the bathroom?

Selected relevant quotes of Delphi consensus process experts:

- "As a participant, I had to walk down a common hallway to use the bathroom (escorted), that felt awkward."
- "Really important that safe and easy access to toilets is had, not leaving the safe cocoon of the treatment room to be thrust into harsh clinical environments."
- "I worked at a site in which the toilet was in the hospital's waiting room, and was shared with the general public. That didn't work well."

Dosing session procedure

Item 8. Number and role of people present

Number and roles of people present, including participants, study staff and informal support.

An individual's interpersonal environment, whether in a mainstream healthcare setting like a hospital or in a recreational or ceremonial space, is known to strongly impact the nature of their experience and its outcomes. The interpersonal environment, which includes the research team, the staff and other patients/participants, as well as carers, friends, and family members, was cited in a qualitative study on hospital in patients' experience as the main source of their sense of safety during their admission (Barrow et al., 2023). In psychedelic research, studies show that group-based psychedelic dosing sessions can instate a sense of intense 'togetherness' and 'shared humanity', otherwise known as '*communitas*', which is related to long-term beneficial outcome, e.g., (Kettner et al., 2019). Given the strong impact of the interpersonal environment on participant outcomes, Item 8 serves to outline who was in the room with participants under the influence of psychedelics, and the role they served.

<u>Suggested reporting questions</u>:

- How many people were present during dosing sessions?
    - How many participants?
    - How many facilitators or guides (per participant)?
    - How many study staff, such as medical staff and researchers?
    - How many informal supports, if any (friends, family, peers)?

<u>Selected relevant quotes of Delphi consensus process experts:</u>

- "Who is present and role of those present."
- "Informal supports around the participant – family, friends – vs. formal supports around the participant – medical providers, therapists etc."
- "How many people in the room during the session? – Both in terms of how many subjects taking the substance (or if it's an individual session), as well as in terms of how many researchers."

Item 9. Positioning

Relative position of people in the room, and what participants were positioned on (e.g., bed, mat, couch).

There is little published in the literature on how individuals' physical positioning may impact psychedelic experiences. Item 9 explores the configuration of the individuals present in the dosing session. It encourages the study team to report on the relative positioning of facilitators to participants, and of participants to each other in the context of group sessions. It also details participants' individual position (e.g., sitting or supine) and what they were positioned on (e.g., a chair or a bed).

Suggested reporting questions:

- How were facilitators positioned relative to the participants?
- If sessions were conducted in groups, what was the participant configuration?
- What were individual participants' position for most of the dosing session? For instance, sitting, supine, or reclined?
- What were participants positioned on? For instance, a bed, sofa, mattress, or rug?
- Could participant change positions throughout the session?

Selected relevant quotes of Delphi consensus process experts:

- "Position of the participants relative to the setting – Fundamental to know who sits where and besides who. This can balance the shape of the space (do a circle in a square room)."
- "Physical placement of therapists relative to client – Important for understanding the level of perceived proximal support for client during their experience."
- "It is important to feel comfortable, safe and be able to choose the position you want to be in throughout the trip. I felt a bit constrained only having a hospital type bed to lie on."

Item 10. Focus and main activities

Focus (internal or external) and main activities of the dosing session.

The focus of a psychedelic dosing session will influence the nature of the activities offered to participants under the influence of psychedelics, and in turn, impact their experience. For instance, a session with an inward focus may encourage participants to pay attention to what arises internally, while lying down, eyes closed, listening to music (Grof, 1980; Johnson et al., 2008). Participants may also be advised to refrain from verbalizing the experience until the end of a dosing session (Gasser et al., 2015). Conversely, in more outward-focusing sessions, participants may be encouraged to verbalize or discuss the experience as it unfolds, as is often the case in MDMA-assisted therapies (Mithoefer, 2017), or to interact with others/their environment through activities like sharing, chanting, dancing, or walking through nature in more traditional and/or group-based contexts.

<u>Suggested reporting questions</u>:

- Did the psychedelic dosing sessions generally have an inward or outward focus?
- If inward, how was this achieved?
- If outward, how was this achieved?

<u>Selected relevant quotes of Delphi consensus process experts:</u>

- "Open or closed nature of activity/ experience in session – freedom to act/ experience in any way versus an agenda set (therapeutic discussion, completing tasks)."
- "General structure and participant activities during dosing sessions (i.e. all eyes-closed music listening vs conversations/sharing vs conducting measurements vs singing/chanting/dancing vs walking through nature, …)."
- "Experience is inevitably greatly affected by whether the experience is social, solo, or solo-but-witnessed/shared."

Item 11. Music or soundscapes

Music or soundscapes that accompanied the dosing experience.

Music is likely the best studied setting variable in psychedelic research. It has been qualified as the 'hidden therapist' for the central role it plays in psychedelic therapy, alluding to how it may help participants work through difficult emotions (Eisner, 1997; Kaelen et al., 2018), and to generally guide the nature of the experience itself. Previous studies suggest that the effects of LSD, among other psychedelics, are amplified by music (Jobst et al., 2021; Preller et al., 2017). Qualitative studies show that participants describe music as conduit that enabled them to surrender to painful memories or emotions (Breeksema et al., 2020). In Western psychedelic research, music is often employed to potentiate psychedelic drug effects, to elicit personally meaningful experiences by intensifying emotions and mental imagery, and to provide non-verbal structure and grounding for the unfolding internal process (Barrett et al., 2018; Kaelen et al., 2018). In traditional indigenous contexts, music is often a live performance, in the form of chants or songs accompanied by instruments, and is considered the conduit through which the psychedelic ritual unfolds (Jerotic et al., 2024; Sabina, 2003).

Suggested reporting questions:

- What sounds or music were employed during dosing sessions?
- Was the music live or recorded? If recorded, is the playlist available?
- Was music played with speakers and/or headphones?
- Were there periods of silence?
- Did participants choose the music? Was it familiar or unfamiliar to them? Could they choose silence?
- What qualities of the music were curated, such as emotionality, lyrics, genre, arc, tempo?

Selected relevant quotes of Delphi consensus process experts:

- "Sound(s) – E.g., silence vs natural/ambient sounds vs music vs other; what types of music (if used) and who selected/controlled it; how music/sounds were heard (headphones vs speakers vs something live); etc. All should be specific enough to support replication."
- "Music has been consistently identified by clinical trial participants as central drivers of their experiences during dosing sessions."
- "Playlist – This needs to prepared with participant(s) in mind and when possible, chosen by a participant. Spaces for silence can also be really important."

Item 12. Interpersonal interventions

Verbal or physical interpersonal interventions used throughout the session, and how consent was obtained.

Interpersonal interventions, whether verbal or physical, are common features of psychedelic-assisted therapy sessions. The use of verbal interventions varies greatly across study groups, depending on the therapeutic model, the substance used, the population treated, and the individual participants/facilitators themselves, potentially ranging from simple 'check-ins' to consistent conversations during dosing sessions.

Physical interventions, including physical touch and bodywork, are sometimes used in psychedelic-assisted therapy. Physical touch may involve facilitators offering to hold participants' hand, foot, or shoulder in moments of distress (Guss et al., 2020; McCabe, 1977), whereas some types of body work involve body contact that is aimed at "intensifying and thereby releasing body tension and pain", by giving subjects something to "push against" (Oehen et al., 2013). Although facilitator-participant touch is thought to play an important role in psychedelic work, from the perspective both of participants (Johnson et al., 2008) and clinicians (e.g., Mithoefer, 2017), there is a lack of research in this area. Of note, this unique practice involves heightened ethical risks, given that psychedelic's mind-altering properties may impair participants' autonomy and capacity to consent to touch (Brennan et al., 2021; Lee et al., 2024). For this reason, and in the context of early and recent instances of sexual violations in the field (McNamee et al., 2023) (MAPS, 2019), it is essential for researchers to outline how consent was obtained and how these ethical risks were mitigated (Butler et al., 2023; Lee et al., 2024). The Yale manual of psilocybin-assisted therapy for depression provides an example of reporting a touch-related consent protocol (Guss et al., 2020).

<u>Suggested reporting questions</u>:

- What types of interpersonal interventions (physical or verbal) were used throughout the session?
- If physical, what types? For instance, physical touch or 'body work'?
- If verbal, what types? For instance, verbal check-ins, instructions, guidance, mantras, or conversations with participants?
- How frequently were these interventions employed?
- How was consent obtained prior to initiating physical interventions?

<u>Selected relevant quotes of Delphi consensus process experts:</u>

- "What the persons involved (guides etc.) are verbally and non-verbally doing (or not doing)."
- "What kind of interaction takes place between the research team and the subjects during the acute effects of the substance? (is there therapeutic intervention? Is there touch? Is the team only supportive in case of difficulties?)"
- "Exploring about touch in consent with the client beforehand."

Item 13. Participant autonomy, control, and agency

Level of participant control and agency over activities and environment of the dosing session.

A survey by Carbonaro et al. (2016) found that individual-led changes (such as changing locations or changing the music environment) can be successful in softening challenging experiences with psilocybin mushrooms. Furthermore, meta-analytic evidence demonstrates that incorporating participant preferences into therapeutic interventions can improve therapeutic outcomes (Kowalski & Mrdjenovich, 2013), including exploring participants' musical preferences in psychedelic-assisted treatments (Garel et al., 2023). On the other hand, clinical trials investigating a particular intervention may not be amenable to personalization (Davis, 2023). Research teams are encouraged to report if and how participants were able to express agency and autonomy during the trial.

There is some overlap between this item and previous items as participant control may entail modifying lighting (Item 5) or music (Item 11), for instance. This item differs from previous items in that it is specifically focused on the ways in which participant control and choice are implemented, rather than the particular contextual element that are being modified. Additionally, it may facilitate the reporting of extra-pharmacological elements that are not specifically reported elsewhere, such as temperature.

Suggested reporting questions:

- How much control did participants have over their movements and behaviors during dosing sessions? For instance, were they encouraged to stay supine or could they change body positions to stand, walk, dance, or change locations?
- How much control did participants have over the physical environment of the dosing sessions? For instance, could they personalize it with some of their own objects or were they involved in room decoration, lighting, temperature, or smells?
- How much control did participants have over their level of sensorial exposures? For instance, could they decide whether or not to employ eyeshades, or the volume of music?

Selected relevant quotes of Delphi consensus process experts:

- "The degree of participant autonomy and control over the experience, including the ability to make choices about their environment and behavior during the experience."
- "Every aspect of the setting should be flexible during the experience. I.e. If the px wants the lights brighter/dimmer, the music louder/softer/off all together, wants to have eyes open/uncovered. Obviously so long as it doesn't endanger them and is feasible to accomplish."
- "The possibility to freely choose one's own activities is crucial. Otherwise, the individual may feel constricted or stressed which is highly unpleasant in psychedelic states."

Item 14. Dosing regimen

Dosing regimen, including drug dose(s), frequency, route of administration and length of the dosing session.

This item encourages the detailed reporting of the dosing regimen employed in a given psychedelic clinical trial. Although details of pharmacological interventions, such as dosages and routes of administration, are already reported in clinical trials as per stadnard practice, this item encourages the reporting of greater details to account for different dosing practices across psychedelic clinical trials. For instance, the length and timing of the psychedelic dosing session.

Suggested reporting questions:

- How long was the dosing session? Did it start in the morning or afternoon?
- What was the drug dose and route of administration? For instance, oral, intramuscular, subcutaneous, sublingual, or inhaled?
- What was the frequency of administration? For instance, a single dose versus multiple doses or optional 'top-ups', and continuous versus intermittent?

Selected relevant quotes of Delphi consensus process experts:

- "I would expect to see items relating the dose (low-high) dose frequency (how many doses on a session-day or weekend) and route of administration."
- "It might be important to understand when the drug(s) was/were administered and how (e.g., a continuous IV dosing of DMT for 1 hour is different than a bolus at time X [...])."
- "The time of day of the trial can influence what a person has already experienced that day, what they have eaten, how tired they are, how present they can be."

Item 15. Medical and experimental procedures or assessments

Medical and experimental procedures or assessments performed during the dosing session.

The heart rate and blood pressure of participants are commonly monitored in the context of psychedelic clinical trials given that most psychedelics can have stimulatory hemodynamic effects. Similarly, drawing blood samples from participants is relatively common in order establish substance pharmacokinetics or the plasma concentration of certain biomarkers in relation to the administration of psychedelic substances (Vogt et al., 2023). Participants in research trials are often also required to complete numerous tasks and questionnaires. These medical/experimental interventions require visible equipment, require time, and ask participants to make various efforts. They may be experienced as invasive or annoying for some or reassuring and meaning-laden for others. Such interventions have recently been shown to influence the course of participants' dosing experience (Roseman et al., 2024).

Suggested reporting questions:

- What medical procedures, if any, were performed during the dosing session? For instance, monitoring vital signs, taking blood samples?
- What experimental assessments, if any, were performed during the dosing session? For instance, visual analogue scales, questionnaires, neuroimaging, or other experimental tasks?
- When were these medical/experimental assessments performed?

Selected relevant quotes of Delphi consensus process experts:

- "Requirement for behavioral or physiological measures (presence of questionnaires, computers, imaging, etc)."
- "Timing of research questionnaires in relation to the dosing."
- "Blood drawing and neuroimaging can be disturbing elements during the experience."

Item 16. Pre- and post-dosing protocol

Activities that took place immediately prior to- or post-dosing, including participant arrival and release conditions..

There have been different approaches to supporting participants logistically, physically, and emotionally immediately prior to and after psychedelic dosing. For instance, participants may receive a detailed overview of the day's activities, have access to quiet spaces for relaxation after the session, or receive assistance in arranging transportation back home at the end of the day. Related activities may include familiarizing participants with the space and objects that will be used during dosing, performing opening or closing rituals, or the provision of food or beverages during actual dosing sessions. This item details how participants were supported on the day of a dosing session prior to beginning the session and after the psychedelic experience has taken place.

Suggested reporting questions:

- What activities took place prior to participants ingesting the psychedelic substance? For instance, a spoken outline of the treatment's schedule, an opening ritual, or a grounding or breathing exercise.
- What activities took place at the end of participants' psychedelic experience? For instance, a closing ritual or a debriefing session.
- How and when were participants deemed safe for discharge home?
- Was food and drink made available to participants before and/or after dosing?

Selected relevant quotes of Delphi consensus process experts:

- "After-care during the 'come down' and beyond."
- "Access to a variety of foods and liquids."
- "Rite for substance use – the rite makes reference to the traditional use and gives relevance to the act itself. Important in placebo and non-placebo groups."

Item 17. Potential disturbances or interruptions

Disturbances or interruptions that may have impacted the quality of the dosing session.

Psychedelic research emphasizes the importance of providing a ‘safe container’ or ‘holding environment’ to individuals under the influence of psychedelic substances (Noorani, 2021). Given the time, space, and budgetary constraints of clinical trials, there are numerous variables that could arise to ‘break’ that sense of containment and negatively impact participants’ experiences, as found in a recent publication (Evans et al., 2023). For instance, within a hospital setting, there are multiple low- and high-frequency sounds from uncontrollable sources, such as ventilation systems, air-conditioning, paging systems, telephones and intercoms (Iyendo, 2016). This noise has been found to negatively impact the mental health and (cognitive) performance of both participants (Ryherd et al., 2008; Short et al., 2011) and staff (Joseph & Ulrich, 2007), and such effects may well be worse under the effects of a psychedelic drug. Similarly, at the end of a session, as scheduled or prematurely, a participant may feel adversely affected by having to return to higher stimulation environments. Given that all such manner of interruptions or disturbances may alter the course of psychedelic experiences (Roseman et al., 2024), item 17 encourages study teams to report instances when dosing environments were sub-optimal due to uncontrollable or unforeseen circumstances.

<u>Suggested reporting questions</u>:

- Where there any disturbances or interruptions during dosing sessions?
    - Unpleasant sensory stimuli, such as noises (machines beeping, traffic, loud chatter), overpowering smells, or unpleasant temperatures?
    - Compromises to participants privacy?
- Did time constraints or other factors cause dosing sessions to be terminated prematurely?

<u>Selected relevant quotes of Delphi consensus process experts:</u>

- “I think this is especially important to report. Since disturbances/interruptions may often be unintentional, it may be helpful to think about how they can be explicitly defined so that researchers/clinicians don’t minimize them.”
- “Interruptions can cause anxiety, which would adversely affect outcome.”
- “No rush/time constraints! [...] I am aware from direct accounts of clinical trial protocols that have interfered with participants process by forcing them to leave the room for example because the “time is up” even though person was still in process.”
- “Privacy – it is very important that the patient knows that he/she can speak loudly (or cry, or scream) without other people hearing or being disturbed.”
- “Minimizing unpleasant or unexpected noises feels important to make pace comfortable and relaxing. Similarly, it’s [...] important not to smell unpleasant! Eg. Strong chemical cleaning products.”

Therapeutic framework and protocol

Item 18. Therapeutic or guiding approach

The therapeutic or guiding approach used throughout the study, if any, with the accompanying manual or protocol.

Clinical trials involving psychedelic-assisted therapy commonly employ a three-phase structure consisting of preparation, dosing, and integration, but the therapeutic frame through which facilitators interact with and guide participants over the course of these sessions is not always specified (Belser & Brennan, 2023). Different therapeutic frameworks emphasize different aspects of the psychedelic experience and the role or significance of the psychotherapeutic interventions. Whereas many research groups evoke a 'non-directive' or 'supportive' stance (Ching et al., 2023), others have published protocols of varying levels of detail for guiding participants in psychedelic-assisted psychotherapy trials. Some examples are the Accept, Commit, Embody (ACE) model (Watts, 2021), the EMBARK model (Brennan & Belser, 2022), the Acceptance and Commitment Therapy (ACT) model (Sloshower et al., 2020), and the Montreal Model of Ketamine (Garel et al., 2023). Several recent reviews have compared the many existing approaches that have been reported (Bathje et al., 2022; Cavarra et al., 2022; Thal et al., 2022).

Suggested reporting questions:

- What therapeutic approach or framework, if any, was used throughout the study? For instance, a 'non-directive'/supportive approach, a custom approach such as Accept, Embody, Connect (ACT); or a manualized psychotherapy such as Acceptance Commitment Therapy (ACT)?
- Were their particular overall characteristics of the therapeutic approach?
    - Standardized or individualized?
    - Based on traditional practices or on previously protocolized psychotherapies?
    - Trauma-informed, transculturally-informed, or participant-centered?
- Is a manual/protocol describing this approach available?

Selected relevant quotes of Delphi consensus process experts:

- "Therapy model used by the team – Relevant as this will be the framework accompanying the psychedelic experience."
- "Info on psychological model/ guiding manual applied. And some info about same thing as above w re to control condition."
- "Mental Health experiences at its core, is based on the ripple effects of trauma (historical, ancestral, racial, ACEs). By having a racial and trauma informed clinician, guide, whoever is hold space for the receiver of the medicine, creates an inclusive setting."
- "Patient centred – taking into account variables such as neurodiversity and being able to adapt setting accordingly."

Item 19. Narrative framing

Framing of the trial intervention by the study team, including the short- and long-term drug effects.

Narratives are intrinsic to patient-clinician interactions (Frank, 2013; Kleinman, 2020). They can the vehicle through which patients understand and communicate their health problems, past histories, and current concerns, and enable clinicians to provide accounts of symptoms and illness that make sense of patients' suffering, clarify its potential course and outcomes, and provide a rationale for specific treatment interventions (Kirmayer et al., 2023). In clinical trial contexts, the way that psychedelic interventions are framed to participants is likely to have powerful effects, as supported by past psychedelic research findings and the broader body of literature showing the influence of patient expectations on clinical outcomes (Aday et al., 2022; Benedetti, 2014; Olson et al., 2020). Different research groups approach psychedelics through different narrative lenses, including mystical, psychotherapeutic, or neuroscientific framings, and may have varying ways of describing their expected effects. Item 19 encourages the study team to reflect on the narrative framing of their study and to report how that may be transmitted to participants.

Suggested reporting questions:

- How was the psychedelic substance and its expected short-term and long-term effects framed to participants?
- Was the psychedelic substance considered as an adjunct to another therapeutic process or rather the core component of the intervention?
- Was there any flexibility in how psychedelic drugs were framed to participants, such as being adapted to their personal beliefs or backgrounds?
- What overarching paradigm guided the study team's approach? For instance, biological, psychological, spiritual, or religious.

Selected relevant quotes of Delphi consensus process experts:

- "Study narrative – the story told about the trial that the participant has been exposed to (e.g. therapeutic, exploratory, promising, scary, etc)."
- "The metaphysical beliefs of the research team – Teams interested in spirituality tend to find the mystical experience importance in predicting outcomes where it is often not the case for teams who do not."
- "Researchers appraisals of the mechanisms and efficacy of psychedelic therapy."

Item 20. Number of sessions

Number and length of preparation, dosing, and integration sessions.

‘Psychedelic-assisted therapies’ are interventions whereby a psychotherapeutic intervention supports and/or is supported by the administration of a psychedelic substance. The psychotherapy literature suggests that there is a ‘dose-response’ curve, whereby more therapy sessions lead to better participants outcomes at least to a point. Indeed, a systematic review found consistent support for a plateauing curvilinear relationship between the number of sessions and the probability of response to treatment, illustrating large incremental gains in the first ~10 sessions of therapy and relatively smaller gains thereafter (Robinson et al., 2020). It is thus crucial for researchers to outline the details of the non-drug intervention sessions, including the length and number of therapy sessions provided either before or after the psychedelic dosing session, often referred to as ‘preparation’ and ‘integration’ respectively.

Suggested reporting questions:

- How many preparation, dosing, and integration sessions did participants receive throughout the trial?
- How long did preparation, dosing, and integration sessions last?

Selected relevant quotes of Delphi consensus process experts:

- “Number of therapy sessions given to prepare participant.”
- “In my research (unpublished), we have found that the dose of therapy (amount of non-drug session therapy hours per medication session) predict clinical symptomatology outcome effect sizes in a review of published psychedelic clinical trials.”

Item 21. Preparation protocol

Activities performed during the preparation sessions.

Participants typically receive ‘preparation’ sessions prior to a psychedelic-based intervention. However, the psychedelic literature remains relatively vague and inconsistent on what these sessions entail (Bathje et al., 2022; Sloshower et al., 2020). Broadly speaking, preparation session are meant to provide contact time between participants and therapists/facilitators, often the same who accompany them during their dosing experience but not always (Rosenblat et al., 2024). These sessions aim to cultivate therapeutic alliances and to prepare the participant for their upcoming experience (Watts & Luoma, 2020). Some more specific aims of preparation sessions cited in the literature include the following: managing participant expectations (Garel et al., 2023), providing psychoeducation regarding the likely effects of the psychedelic substances (Cavarra et al., 2022), and also discussing participant fears, intentions, and motivations (Sloshower et al., 2020). The importance of adequate preparation activities has been highlighted in studies showing significant associations between preparation and mental health outcomes e.g. (Perkins et al., 2021), and from participants themselves who noted the importance of preparatory sessions to their personal experiences (Breeksema et al., 2020). A novel tool – the Psychedelic Preparedness Scale – was recently developed to assess whether a person is an adequately prepared psychological state to benefit from/not be harmed by an upcoming psychedelic experience (McAlpine et al., 2024).

Suggested reporting questions:

- What activities were performed during preparation sessions? For instance:
    - Taking participants’ past medical/psychological histories;
    - Surveying participants’ past experience with psychedelic substances;
    - Encouraging participants to set intentions;
    - Discussing participants’ expectations;
    - Addressing questions and concerns;
    - Providing psychoeducation;
    - Familiarizing participants with the dosing space;
    - Q&A sessions with family members and friends;
    - Encouraging participants to engage in independent activities or behaviors like yoga, meditation, or time in nature;
    - Encouraging participants to not engage in certain activitie like spending time online or eating certain foods.
- Who facilitated the preparation sessions?

Selected relevant quotes of Delphi consensus process experts:

- “Types and extent of preparation activities – depending on background of the participant/session this will have a crucial impact on acute and long-term effects.”
- “Details of the preparatory session – Can influence expectations and enhance placebo effect.”
- “Whether participants are encouraged to set an intention – Speaks to the therapeutic framework, as well as how the setting might cultivate a specific set-in participants.”

Item 22. Integration protocol

Activities performed during the integration sessions.

‘Integration’ sessions occur following a psychedelic experience, and generally serve to support insights that emerged from the experience to promote lasting changes in the participant’s life. Earleywine and colleagues have proposed the following definition of integration, based on interviews with 30 integration therapists: “Integration is a bridge from the psychedelic experience to everyday life that helps clients make sense of their experience in a personalized way, leading to lasting behavior change and a sense of wholeness or completion” (Earleywine et al., 2022). Bathje and collegues (2022) further suggest that integration “is a process in which a person revisits and actively engages in making sense of, working through, translating and processing the content of their psychedelic experiences”. In their systematic review, the authors identified a total of 10 models being used for integration in addition to an exhaustive list of specific integration practices. These include: taking part in creative endeavors (drawing, music), practicing yoga or mindfulness meditation, lifestyle changes (diet, journaling, hobbies), spending time in nature, and community-based activities (Bathje et al., 2022). Such practices may directly influence outcomes. For instance, a study found that participating in body therapies (like yoga and tai-chi) was associated with greater psychological wellbeing and growth (Perkins et al., 2021). Given the large array of goals and activities that could fall under ‘integration’, researchers are encouraged to standardize and detail their own integration protocol (Frymann et al., 2022).

Suggested reporting questions:

- What activities were performed during integrations sessions? For instance:
    - Generating a written narrative of the experience;
    - Making meaning from the experience through a specific art form, such as drawing, painting, poetry, sculpting, dancing, or musical composition;
    - Revisiting the experience with mnemonic aids such as re-listening to music from the dosing session;
    - Addressing challenging aspects of the psychedelic experiences;
    - Identifying lifestyle targets for change;
    - Discussing or socializing with other participants.
- Who facilitated the integration sessions?

Selected relevant quotes of Delphi consensus process experts:

- “Details of integration sessions.”
- “How is the experience interpreted and by whom?”
- “Integration framework (i.e.: does the facilitator or researcher ,”set up” the experience by explaining it to the participant through a particular framework, such as mysticism, somatic experiencing, new age spirituality, etc.).”

Item 23. Additional support or follow-up

Formal or informal support or follow-up offered to participants after the end of the trial, including in the case of adverse events.

Participants may be destabilized, disappointed, or otherwise negatively impacted by psychedelic experiences (Breeksema et al., 2022). Study teams should therefore detail how they manage such outcomes (Evans, 2024). Beyond safety considerations, optional or routine follow-up appointments may also significantly influence treatment outcomes: a meta-analysis examining antidepressant drugs for major depressive disorder found that additional follow-up assessments (lasting approximately 30 minutes) were correlated with a cumulative and proportional reduction of depressive symptoms, accounting for 34-44% of the effects of both placebo and active treatment groups (Posternak & Zimmerman, 2007). For these reasons, the details of follow-up visits are critical to report. Additionally, participants may pursue informal forms of support or community after a psychedelic intervention, such as peer or advocacy psychedelic groups. Whether participants are formally referred to such resources, or pursue them independently, these activities should also be reported as they may impact subsequent outcomes (Noorani et al., 2023).

Suggested reporting questions:

- Did participants receive any formal additional support or follow-up? For instance, additional therapy sessions or medical check-ins?
    - Were this routine or provided on a as-needed basis?
    - How many such sessions did participants receive, and long were these sessions?
- Did participants receive additional support from informal groups, either on their own accord or based on recommendations of the study team?
- Did participants maintain formal or informal contact with the study team following the end of the trial?
- How were post-trial adverse events approached?

Selected relevant quotes of Delphi consensus process experts:

- “Continuity of care from not only the therapeutic team, but the clinical team doing medical checks, IV lines etc.”
- “Social supports of participants (including group/individual therapy and support groups).”
- “Availability of continued therapy – Being able to integrate experiences within a broader and sustained therapeutic framework is very important (vs falling into a ‘black hole’).”

Item 24. Study personnel qualifications

The credentials, training, and expertise of personnel providing the study intervention or care.

The psychotherapy literature suggests complex and multifaceted links between therapist experience levels and qualifications with treatment outcomes (Erekson et al., 2017; Stein & Lambert, 1984), though psychiatric outcomes consistently vary between therapists and prescribers (McKay et al., 2006; Wampold & Imel, 2015). Qualifications and skillsets of study teams may be particularly relevant in psychedelic therapies given the marked diversity and intensity of their effects. For instance, multidisciplinary teams with diverse skillsets and backgrounds may be more likely to provide adequate care to participants of diverse backgrounds (Blainey, 2021; Williams et al., 2020). Furthermore, given the rapidly-evolving state of knowledge regarding psychedelic drugs (Heal et al., 2023), the levels of expertise and experience that team members have with psychedelic therapies may vary widely, and thus warrant reporting.

Suggested reporting questions:

- What were the study personnel's credentials?
- Did study personnel undergo any particular training for the purpose of the study?
- What is level of experience guiding psychedelic experiences did the facilitators have?

Selected relevant quotes of Delphi consensus process experts:

- "Presence of a guide and/or sitter, including (if applicable) the role they play, their background/training, and whether they were familiar to the participant, and how."
- "Guide skill mix – to have multi-disciplinary mix between guides to allow for multiple points of views and trainings. Importantly this does not need to be therapists. Nurses have excellent skills in this area."
- "Profile of therapists (professional degree and therapy school affiliation)."

Item 25. Cultural competence and safety

Study team's level of cultural competence and efforts towards cultural safety.

'Cultural competence' is defined as the ability to care for patients/participants with diverse values, beliefs, and behaviors, by tailoring interventions to their social, cultural and linguistic needs (Betancourt et al., 2003). Cultural competence can be fostered at the level of the institution, the professionals within it, and the intervention provided itself (Kirmayer, 2012). In psychiatry specifically, good clinical practice may require cultural competence such that practitioners have a responsibility to seek out training to address personal biases and develop skills in working in cross-cultural therapeutic relationships (Kirmayer et al., 2012). 'Cultural safety' goes beyond the concept of cultural competence and is predicated on increasing health access and equity for minority/oppressed groups by urging individual professionals to examine their own forms of privilege and address power imbalances that are inherent to therapeutic encounters (Lokugamage et al., 2023). Currently, psychedelic trials are disproportionately comprised of white participants (Hughes & Garcia-Romeu, 2024; Michaels et al., 2018) and there is a lack of ethno-racial diversity among psychedelic researchers and therapists (Williams & Labate, 2020). If equity-oriented approaches are not utilized in the development and administration of psychedelic-assisted therapies, these therapies might become least accessible to those who most need them, as per the 'Inverse Care Law' (Hart, 1971; Rea & Wallace, 2021). Item 20 thus encourages study teams to reflect and report on how the trial was made accessible, safe, and relevant to marginalized groups, if at all. This may include specific trainings, recruitment strategies, or other efforts to ensure the diversity and cultural safety of the study team and the trial sample (Williams et al., 2020).

Suggested reporting questions:

- How was cultural safety fostered at the institutional level, in the research team, and in the therapeutic container?
- Were there culturally sensitive practices specifically incorporated into the design of the trial and its therapeutic approach?
- Did study team members undergo anti-oppressive or similar trainings?
- Were demographic-specific or culturally-tailored recruitment or enrollment strategies used to ensure diversity of the trial's sample?

Selected relevant quotes of Delphi consensus process experts:

- "Cultural safety - guides being specifically trained and being aware of their own limitations when being able to 'hold' specific aspects of racial trauma. There is a complete lack of training in this area."
- "If patient is part of minority/marginalized population, having someone with lived experience can be helpful // might not be practical, but affects perceived safety/connection of participant."
- "The history and practice of psychedelics is ancestral, predominantly from indigenous, black, and other racialized communities, and may not necessarily be from the historical lineage of white people."

Subjective experiences

Item 26. Therapeutic alliance

Therapeutic alliance between participants and facilitators throughout the intervention.

Therapeutic alliance refers to the presence of a collaborative relationship between patients and practitioners that includes common treatment goals and a positive emotional bond (Bordin, 1979). Therapeutic alliances have been found to strongly influence therapeutic outcomes across fields and interventions in healthcare (Priebe et al., 2019). Indeed, improving patient-clinician relationships can significantly improve both objective and subjective health outcomes in a variety of disease states (Kelley et al., 2014), and increased clinician empathy can positively impact patients' levels of pain, anxiety and satisfaction (Howick et al., 2018). In psychedelic research, therapeutic alliance has been linked to outcomes for psilocybin-assisted therapy for depression (Levin et al., 2024; Murphy et al., 2022) and MDMA-assisted therapy for PTSD (Zeifman et al., 2024). Given its demonstrated impact on therapeutic outcomes, item 26 encourages study teams to report on therapeutic alliances between participants and facilitators in clinical trials with psychedelics, by describing how it was fostered and/or by reporting how it was measured.

<u>Suggested reporting questions</u>:

- How were strong therapeutic alliances cultivated in the study?
- Were any interviews conducted, or specific tools employed, to evaluate therapeutic alliances?

<u>Suggested measuring tools:</u>

- The STAR scale (McGuire-Snieckus et al., 2007)
- The Working Alliance Inventory-Short Revised (WAI-SR) (Hatcher & Gillaspy, 2006)
- The Working Alliance Inventory (Horvath & Greenberg)

<u>Selected relevant quotes of Delphi consensus process experts:</u>

- "The nature of the therapeutic relationship can be complex and multi-faceted, and it can have a profound impact on the quality and depth of the psychedelic experience."
- "The therapeutic alliance and other relationships or familiarity with those who enter the space is often very significant for the quality of the experience."
- "Participant perception of empathy, respect and genuineness of therapists."

Item 27. Trust

Participant's level of trust throughout the intervention.

Trust is often cited as fundamental to participants 'letting go' and embracing the process of psychedelic experiences and therapies (Watts & Luoma, 2020). The concept of trust may relate to a participant's trust in their environment, in the people around them including their guide(s) (McAlpine et al., 2024; Skiles et al., 2023), and in the substance they were administered. In this way, it overlaps partially with the concept of therapeutic alliance but is more generalized, going beyond the strength of therapeutic working relationships. A study by Carbonaro et al. (2016) showed that the trust of others present during a psychedelic experience was conducive to individuals having a positive experience with psilocybin mushrooms. Despite its perceived importance, 'trust' remains a relatively poorly operationalized concept. Crits-Cristoph and colleagues recently published a preliminary new scale to assess the level of trust between a patient and clinician (Crits-Christoph et al., 2019), but there is no scale as of yet to measure a broader sense of trust during psychedelic experiences.

Suggested reporting questions:

- How was participant trust for the environment, themselves, others, and the psychedelic substance cultivated in the study?
- Were any interviews conducted, or specific tools employed, to evaluate participant levels of trust?

Suggested measuring tools:

- No specific tools have been developed to measure participant trust in psychedelic clinical trials.
- Trust could potentially be evaluated by adapting the Setting Questionnaire for the Ayahuasca Experience (SQAE) (Pontual et al., 2021).

Selected relevant quotes of Delphi consensus process experts:

- "For the participant to feel able to fully let go, they need to have trust in themselves to be able to face and cope with whatever may arise (valuable to build in prep work for this beforehand), to trust in the psychedelic substance they have taken - that it will hopefully present valuable insight, and that any difficulties will be navigable (potentially with help), and, finally, to trust in the team around them, to support them, help them through any difficulties and metaphorically stand alongside them throughout."
- "Many dimensions of trust--in relationship with self and guides."
- "Trust essential to smooth flow of experience for everyone."
- "The overarching themes of safety and trust just cannot be overemphasised. Safety and trust in the therapeutic alliance, the physical space (not only during dosing, but once discharged onto the wards), and the preparation required to create an optimal container for the experience."

Item 28. Physical comfort

Participant's level of physical comfort during the dosing session.

Psychedelics are known to produce alterations in low-level sensorium, for instance leading to changes in vision, proprioception and bodily felt sensations (Aqil & Roseman, 2023; Herzog et al., 2023; Ho et al., 2020; Mastinu et al., 2023), and individuals under the influence of psychedelics are also known to be particularly sensitive to their environment. Special attention is thus required to a sense of physical comfort in individuals under the influence of psychedelics, especially for the dosing sessions that typically last multiple hours. In a study on challenging experiences, 75% of participants reported that a sense of physical comfort was conducive to having a positive psilocybin mushroom experience, and was negatively correlated with the degree or length of a negative experience (Carbonaro et al., 2016). Researchers are thus encouraged to report on participants' level of perceived physical comfort during the dosing sessions of their clinical trials.

Suggested reporting questions:

- How was participant physical comfort during dosing sessions ensured in the study?
- Were any interviews conducted, or specific tools employed, to evaluate participant levels of physical comfort?

Suggested measuring tool:

- No specific tools have been developed to measure participant physical comfort in psychedelic clinical trials.
- Physical comfort could potentially be evaluated by adapting the Setting Questionnaire for the Ayahuasca Experience (SQAE) (Pontual et al., 2021).

Selected relevant quotes of Delphi consensus process experts:

- "General comfort, i.e. temperature of the room, air quality, comfortable furniture etc. px must be able to feel relaxed and at ease."
- "Participant comfort with the environment - Apprehension or discomfort with the setting is a known predictor of adverse outcomes with psychedelics."
- "There is nothing worse than being uncomfortable while in a psychedelic session - it can dominate and colour the entire experience."

Item 29. Physical safety

Participant's sense of physical safety during the dosing session.

Psychedelic substances carry a risk profile related to their impacts on individuals' physiology, behavior, and cognition. Physiologically, they are generally known to increase heart rate and blood pressure (Wsół, 2023). Other common side effects include nausea, headaches and anxiety (Romeo et al., 2024). Participants under the influence of psychedelics are also more physically vulnerable due to the impacts of psychedelics on cognition and reasoning abilities (Basedow et al., 2024). In unprepared individuals, the intensity of the emotional and sensorial experience under psychedelics have at times precipitated dangerous behaviors and other serious safety concerns (Strassman, 1984). Although these have been found to be rare in controlled settings (Johnson et al., 2008), there is some concern that physical safety concerns have been under-reported in the existing psychedelic literature (Hinkle et al., 2024).

For this reason, psychedelics generally require supervision and care to ensure their safety. Most psychedelic clinical trials enact measures to ensure participants' physical safety include choosing safe locations, monitoring vital signs, and having readily accessible of rescue medication in the advent of strong adverse reactions (e.g. Guss et al., 2020). While these safety practices are already reported in clinical research as per current norms, Item 29 focuses on the subjective experience of safety of study participants. I.e., how they perceive their physical safety during the dosing sessions of a clinical trials.

Suggested reporting questions:

- How was a sense of physical safety during dosing sessions instilled in participants of the study?
- Were any interviews conducted, or specific tools employed, to evaluate participant levels of perceived physical safety?

Suggested measuring tool:

- No specific tools have been developed to measure participant physical safety in psychedelic clinical trials.

Selected relevant quotes of Delphi consensus process experts:

- "Having a psychedelic experience makes you very vulnerable. You need to feel safe from the outside world. You also need to feel that you will be OK if you have a bad trip."
- "Safety back up system, medical, social support outside the room."
- "Undergoing a trip is scary as you can go to dark places, so you need to feel totally safe."

Item 30. Psychological and cultural safety

Participant's sense of psychological and cultural safety with the people present during the dosing session.

A sense of psychological safety, including culturally and interpersonally, is key to deriving the benefits of psychedelic substances (Carbonaro et al., 2016; McAlpine et al., 2024; Phelps & Henry, 2021). Whereas Item 8 lists the people present and their role, and Item 26 evaluates the Therapeutic alliance between the participant and their guide(s), Item 30 aims to reflect the extent of safety and respect actually experienced by individual participants during a psychedelic clinical trial. For instance, the study team may have emphasized the training of their staff to foster cultural competence and safety, but Item 30 examines whether participants actually felt safe in their cultural identity at the time of dosing (Taylor, 2017).

Suggested reporting questions:

- How was a sense of psychological and cultural safety during dosing sessions instilled in participants of the study?
- Were any interviews conducted, or specific tools employed, to evaluate aspects of perceived psychological and cultural safety in the study? For instance, that participants:
    - Felt like their boundaries were respected;
    - Felt like their identity was welcomed and respected;
    - Felt a sense of familiarity and safety with the people present;
    - Felt like their cultural background was considered and respected.

Suggested measuring tool:

- No specific tools have been developed to measure participant psychological and cultural safety in psychedelic clinical trials.

Selected relevant quotes of Delphi consensus process experts:

- "Social safety is difficult to assess and differently constructed and experienced by different people, but is an essential variable, especially for QTBIPOC folks. There are many anecdotes of queer participants not feeling "safe" in cis-heteronormative psychedelic clinical settings."
- "The px should feel comfortable, safe and relaxed with the people accompanying them during the experience."
- "Implicit ideological orientations- intentions/expectations/cosmology- of embedded social environment/microculture and their fit with the individual - The degree of social comfort will be related to the ideological fit between the individual and social environment."
- "Familiarity- foreign country/language/culture? - Cultural familiarity is a subset of social environment fit."

References

Aday, J. S., Heifets, B. D., Pratscher, S. D., Bradley, E., Rosen, R., & Woolley, J. D. (2022). Great Expectations: recommendations for improving the methodological rigor of psychedelic clinical trials. *Psychopharmacology (Berl)*, *239*(6), 1989-2010. https://doi.org/10.1007/s00213-022-06123-7

Aqil, M., & Roseman, L. (2023). More than meets the eye: The role of sensory dimensions in psychedelic brain dynamics, experience, and therapeutics. *Neuropharmacology*, *223*, 109300.

Barrett, F. S., Preller, K. H., & Kaelen, M. (2018). Psychedelics and music: neuroscience and therapeutic implications. *International Review of Psychiatry*, *30*(4), 350-362.

Barrow, E., Lear, R. A., Morbi, A., Long, S., Darzi, A., Mayer, E., & Archer, S. (2023). How do hospital inpatients conceptualise patient safety? A qualitative interview study using constructivist grounded theory. *BMJ Quality & Safety*, *32*(7), 383-393.

Basedow, L. A., Majić, T., Hafiz, N. J., Algharably, E. A., Kreutz, R., & Riemer, T. G. (2024). Cognitive functioning associated with acute and subacute effects of classic psychedelics and MDMA-a systematic review and meta-analysis. *Scientific reports*, *14*(1), 14782.

Bathje, G. J., Majeski, E., & Kudowor, M. (2022). Psychedelic integration: An analysis of the concept and its practice. *Front Psychol*, *13*, 824077. https://doi.org/10.3389/fpsyg.2022.824077

Benedetti, F. (2014). Placebo effects: from the neurobiological paradigm to translational implications. *Neuron*, *84*(3), 623-637.

Betancourt, J. R., Green, A. R., Carrillo, J. E., & Owusu Ananeh-Firempong, I. (2003). Defining cultural competence: a practical framework for addressing racial/ethnic disparities in health and health care. *Public health reports*.

Blainey, M. G. (2021). *Christ Returns from the Jungle: Ayahuasca Religion as Mystical Healing*. State University of New York Press.

Bordin, E. S. (1979). The generalizability of the psychoanalytic concept of the working alliance. *Psychotherapy: Theory, research & practice*, *16*(3), 252.

Breeksema, J. J., Kuin, B. W., Kamphuis, J., van den Brink, W., Vermetten, E., & Schoevers, R. A. (2022). Adverse events in clinical treatments with serotonergic psychedelics and MDMA: A mixed-methods systematic review. *Journal of psychopharmacology*, *36*(10), 1100-1117. https://journals.sagepub.com/doi/pdf/10.1177/02698811221116926

Breeksema, J. J., Niemeijer, A. R., Krediet, E., Vermetten, E., & Schoevers, R. A. (2020). Psychedelic treatments for psychiatric disorders: A systematic review and thematic synthesis of patient experiences in qualitative studies. *CNS drugs*, *34*, 925-946.

Brennan, W., & Belser, A. B. (2022). Models of psychedelic-assisted psychotherapy: A contemporary assessment and an introduction to EMBARK, a transdiagnostic, trans-drug model. *Frontiers in Psychology*, *13*, 866018.

Brennan, W., Jackson, M. A., MacLean, K., & Ponterotto, J. G. (2021). A qualitative exploration of relational ethical challenges and practices in psychedelic healing. *Journal of Humanistic Psychology*, 00221678211045265.

Butler, J. A., Herzberg, G., & Miller, R. L. (2023). *Integral psychedelic therapy : the non-ordinary art of psychospiritual healing* (1st ed.). Routledge. https://www.vlebooks.com/vleweb/product/openreader?id=none&isbn=9781000912111

Carbonaro, T. M., Bradstreet, M. P., Barrett, F. S., MacLean, K. A., Jesse, R., Johnson, M. W., & Griffiths, R. R. (2016). Survey study of challenging experiences after ingesting psilocybin mushrooms: Acute and enduring positive and negative consequences. *J Psychopharmacol*, *30*(12), 1268-1278. https://doi.org/10.1177/0269881116662634

Cavarra, M., Falzone, A., Ramaekers, J. G., Kuypers, K. P., & Mento, C. (2022). Psychedelic-assisted psychotherapy—A systematic review of associated psychological interventions. *Frontiers in Psychology*, *13*, 887255.

Ching, T., Grazioplene, R., Pittenger, C., & Kelmendi, B. (2023). Yale program for psychedelic science (YPPS) manual for psilocybin combined with non-directive support in the treatment of OCD.

Cooley, S. J., Jones, C. R., Kurtz, A., & Robertson, N. (2020). 'Into the wild': A meta-synthesis of talking therapy in natural outdoor spaces. *Clinical Psychology Review*, *77*, 101841.

Crits-Christoph, P., Rieger, A., Gaines, A., & Gibbons, M. B. C. (2019). Trust and respect in the patient-clinician relationship: preliminary development of a new scale. *BMC psychology*, *7*, 1-8.

Davis, O. (2023). Autonomy and autoheteronomy in psychedelically assisted psychotherapy. In *New Interdisciplinary Perspectives On and Beyond Autonomy*. Taylor & Francis.

Earleywine, M., Low, F., Lau, C., & De Leo, J. (2022). Integration in psychedelic-assisted treatments: Recurring themes in current providers' definitions, challenges, and concerns. *Journal of Humanistic Psychology*, 00221678221085800.

Eisner, B. (1997). Set, setting, and matrix. *J Psychoactive Drugs*, *29*(2), 213-216. https://doi.org/10.1080/02791072.1997.10400190

Erekson, D. M., Janis, R., Bailey, R. J., Cattani, K., & Pedersen, T. R. (2017). A longitudinal investigation of the impact of psychotherapist training: Does training improve client outcomes? *Journal of Counseling Psychology*, *64*(5), 514.

Evans, J., Robinson, O. C., Argyri, E. K., Suseelan, S., Murphy-Beiner, A., McAlpine, R., Luke, D., Michelle, K., & Prideaux, E. (2023). Extended difficulties following the use of psychedelic drugs: A mixed methods study. *PLoS One*, *18*(10), e0293349.

Fischer, R., Hill, R., & Warshay, D. (1969). Effects of the psychodysleptic drug psilocybin on visual perception. Changes in brightness preference. *Experientia*, *25*, 166-169.

Frank, A. W. (2013). *The wounded storyteller: Body, illness & ethics*. University of Chicago Press.

Frymann, T., Whitney, S., Yaden, D. B., & Lipson, J. (2022). The psychedelic integration scales: Tools for measuring psychedelic integration behaviors and experiences. *Frontiers in Psychology*, *13*, 863247.

Gandy, S., Forstmann, M., Carhart-Harris, R. L., Timmermann, C., Luke, D., & Watts, R. (2020). The potential synergistic effects between psychedelic administration and nature contact for the improvement of mental health. *Health Psychology Open*, *7*(2), 2055102920978123.

Garcia-Romeu, A., & Richards, W. A. (2018). Current perspectives on psychedelic therapy: use of serotonergic hallucinogens in clinical interventions. *Int Rev Psychiatry*, *30*(4), 291-316. https://doi.org/10.1080/09540261.2018.1486289

Garel, N., Drury, J., Thibault Levesque, J., Goyette, N., Lehmann, A., Looper, K., Erritzoe, D., Dames, S., Turecki, G., Rej, S., Richard-Devantoy, S., & Greenway, K. T. (2023). The Montreal model: an integrative biomedical-psychedelic approach to ketamine for severe treatment-resistant depression. *Front Psychiatry*, *14*, 1268832. https://doi.org/10.3389/fpsyt.2023.1268832

Gasser, P., Kirchner, K., & Passie, T. (2015). LSD-assisted psychotherapy for anxiety associated with a life-threatening disease: a qualitative study of acute and sustained subjective effects. *J Psychopharmacol*, *29*(1), 57-68. https://doi.org/10.1177/0269881114555249

Grof, S. (1980). LSD Psychotherapy. The healing potencial of psychedelic medicine. In: Hunter House Publishers, Alameda California.

Guss, J., Krause, R., & Sloshower, J. (2020). The Yale manual for psilocybin-assisted therapy of depression (using acceptance and commitment therapy as a therapeutic frame).

Hart, J. T. (1971). The inverse care law. *The Lancet*, *297*(7696), 405-412.

Hartogsohn, I. (2017). Constructing drug effects: A history of set and setting. *Drug Science, Policy and Law*, *3*, 2050324516683325.

Hatcher, R. L., & Gillaspy, J. A. (2006). Development and validation of a revised short version of the Working Alliance Inventory. *Psychotherapy research*, *16*(1), 12-25.

Heal, D. J., Smith, S. L., Belouin, S. J., & Henningfield, J. E. (2023). Psychedelics: threshold of a therapeutic revolution. In (Vol. 236, pp. 109610): Elsevier.

Herzog, R., Mediano, P. A., Rosas, F. E., Lodder, P., Carhart-Harris, R., Perl, Y. S., Tagliazucchi, E., & Cofre, R. (2023). A whole-brain model of the neural entropy increase elicited by psychedelic drugs. *Scientific reports*, *13*(1), 6244.

Hinkle, J. T., Graziosi, M., Nayak, S. M., & Yaden, D. B. (2024). Adverse events in studies of classic psychedelics: A systematic review and meta-analysis. *JAMA Psychiatry*.

Ho, J. T., Preller, K. H., & Lenggenhager, B. (2020). Neuropharmacological modulation of the aberrant bodily self through psychedelics. *Neuroscience & Biobehavioral Reviews*, *108*, 526-541.

Horvath, A., & Greenberg, L. S.(1989). Develipment and validation of WAI. *Journal of Counseling Psychology*, *36*, 223-233.

Howick, J., Moscrop, A., Mebius, A., Fanshawe, T. R., Lewith, G., Bishop, F. L., Mistiaen, P., Roberts, N. W., Dieninytė, E., & Hu, X.-Y. (2018). Effects of empathic and positive communication in healthcare consultations: a systematic review and meta-analysis. *Journal of the Royal Society of Medicine*, *111*(7), 240-252.

Hughes, M. E., & Garcia-Romeu, A. (2024). Ethnoracial inclusion in clinical trials of psychedelics: a systematic review. *EClinicalMedicine*, *74*.

Iyendo, T. O. (2016). Exploring the effect of sound and music on health in hospital settings: A narrative review. *Int J Nurs Stud*, *63*, 82-100. https://doi.org/10.1016/j.ijnurstu.2016.08.008

Jerotic, K., Vuust, P., & Kringelbach, M. L. (2024). Psychedelia: The interplay of music and psychedelics. *Annals of the New York academy of sciences*, *1531*(1), 12-28. https://doi.org/10.1111/nyas.15082

Jobst, B. M., Atasoy, S., Ponce-Alvarez, A., Sanjuán, A., Roseman, L., Kaelen, M., Carhart-Harris, R., Kringelbach, M. L., & Deco, G. (2021). Increased sensitivity to strong perturbations in a whole-brain model of LSD. *Neuroimage*, *230*, 117809.

Johnson, M., Richards, W., & Griffiths, R. (2008). Human hallucinogen research: guidelines for safety. *J Psychopharmacol*, *22*(6), 603-620. https://doi.org/10.1177/0269881108093587

Johnson, M. W. (2020). Consciousness, religion, and gurus: pitfalls of psychedelic medicine. *ACS pharmacology & translational science*, *4*(2), 578-581.

Joseph, A., & Ulrich, R. (2007). Sound control for improved outcomes in healthcare settings. *The Center for Health Design*, *4*, 2007.

Kaelen, M., Giribaldi, B., Raine, J., Evans, L., Timmerman, C., Rodriguez, N., Roseman, L., Feilding, A., Nutt, D., & Carhart-Harris, R. (2018). The hidden therapist: evidence for a central role of music in psychedelic therapy. *Psychopharmacology (Berl)*, *235*(2), 505-519. https://doi.org/10.1007/s00213-017-4820-5

Kelley, J. M., Kraft-Todd, G., Schapira, L., Kossowsky, J., & Riess, H. (2014). The influence of the patient-clinician relationship on healthcare outcomes: a systematic review and meta-analysis of randomized controlled trials. *PLoS One*, *9*(4), e94207. https://doi.org/10.1371/journal.pone.0094207

Kettner, H., Gandy, S., Haijen, E. C., & Carhart-Harris, R. L. (2019). From egoism to ecoism: Psychedelics increase nature relatedness in a state-mediated and context-dependent manner. *International journal of environmental research and public health*, *16*(24), 5147.

Kirmayer, L. J. (2012). Rethinking cultural competence. In (Vol. 49, pp. 149-164): Sage Publications Sage UK: London, England.

Kirmayer, L. J., Fung, K., Rousseau, C., Lo, H. T., Menzies, P., Guzder, J., & McKenzie, K. (2012). Guidelines for training in cultural psychiatry. *Canadian Journal of Psychiatry*, *57*(3), 1-16.

Kirmayer, L. J., Gómez-Carrillo, A., Sukhanova, E., & Garrido, E. (2023). Narrative Medicine. *Person Centered Medicine*, 235-255.

Kleinman, A. (2020). *The illness narratives: Suffering, healing, and the human condition*. Basic books.

Kometer, M., & Vollenweider, F. X. (2018). Serotonergic hallucinogen-induced visual perceptual alterations. *Behavioral neurobiology of psychedelic drugs*, 257-282.

Kong, Z., Liu, Q., Li, X., Hou, K., & Xing, Q. (2022). Indoor lighting effects on subjective impressions and mood states: A critical review. *Building and Environment*, *224*, 109591.

Kowalski, C. J., & Mrdjenovich, A. J. (2013). Patient preference clinical trials: why and when they will sometimes be preferred. *Perspectives in biology and medicine*, *56*(1), 18-35.
Langlitz, N. (2013). *Neuropsychedelia: The revival of hallucinogen research since the decade of the brain*. Univ of California Press.
Lawlor, D. (2013). Returning to Wirikuta: The Huichol and their sense of place. *European Journal of Ecopsychology*, *4*, 19-31.
Lee, A., Rosenbaum, D., & Buchman, D. Z. (2024). Informed Consent to Psychedelic-Assisted Psychotherapy: Ethical Considerations. *The Canadian Journal of Psychiatry*, *69*(5), 309-313.
Levin, A. W., Lancelotta, R., Sepeda, N. D., Gukasyan, N., Nayak, S., Wagener, T. L., Barrett, F. S., Griffiths, R. R., & Davis, A. K. (2024). The therapeutic alliance between study participants and intervention facilitators is associated with acute effects and clinical outcomes in a psilocybin-assisted therapy trial for major depressive disorder. *PLoS One*, *19*(3), e0300501.
Loizaga-Velder, A., & Verres, R. (2014). Therapeutic effects of ritual ayahuasca use in the treatment of substance dependence—qualitative results. *Journal of Psychoactive Drugs*, *46*(1), 63-72.
Lokugamage, A. U., Rix, E. L., Fleming, T., Khetan, T., Meredith, A., & Hastie, C. R. (2023). Translating cultural safety to the UK. *Journal of medical ethics*, *49*(4), 244-251.
Mastinu, A., Anyanwu, M., Carone, M., Abate, G., Bonini, S. A., Peron, G., Tirelli, E., Pucci, M., Ribaudo, G., & Oselladore, E. (2023). The bright side of psychedelics: latest advances and challenges in neuropharmacology. *International Journal of Molecular Sciences*, *24*(2), 1329.
McAlpine, R. G., Blackburne, G., & Kamboj, S. K. (2024). Development and psychometric validation of a novel scale for measuring 'psychedelic preparedness'. *Scientific reports*, *14*(1), 1-15.
McCabe, O. L. (1977). Psychedelic drug crises: toxicity and therapeutics. *Journal of Psychedelic Drugs*, *9*(2), 107-121.
McGuire-Snieckus, R., McCABE, R., Catty, J., Hansson, L., & Priebe, S. (2007). A new scale to assess the therapeutic relationship in community mental health care: STAR. *Psychological medicine*, *37*(1), 85-95.
McKay, K. M., Imel, Z. E., & Wampold, B. E. (2006). Psychiatrist effects in the psychopharmacological treatment of depression. *Journal of affective disorders*, *92*(2-3), 287-290.
McNamee, S., Devenot, N., & Buisson, M. (2023). Studying harms is key to improving psychedelic-assisted therapy—participants call for changes to research landscape. *JAMA Psychiatry*, *80*(5), 411-412.
Michaels, T. I., Purdon, J., Collins, A., & Williams, M. T. (2018). Inclusion of people of color in psychedelic-assisted psychotherapy: a review of the literature. *BMC Psychiatry*, *18*(1), 245. https://doi.org/10.1186/s12888-018-1824-6
Murphy, R., Kettner, H., Zeifman, R., Giribaldi, B., Kartner, L., Martell, J., Read, T., Murphy-Beiner, A., Baker-Jones, M., & Nutt, D. (2022). Therapeutic alliance and rapport modulate responses to psilocybin assisted therapy for depression. *Frontiers in Pharmacology*, *12*, 788155.
Noorani, T. (2021). Containment matters: Set and setting in contemporary psychedelic psychiatry. *Philosophy, Psychiatry, & Psychology*, *28*(3), 201-216.
Noorani, T., Bedi, G., & Muthukumaraswamy, S. (2023). Dark loops: contagion effects, consistency and chemosocial matrices in psychedelic-assisted therapy trials. *Psychological medicine*, *53*(13), 5892-5901.
Oehen, P., & Gasser, P. (2022). Using a MDMA-and LSD-group therapy model in clinical practice in Switzerland and highlighting the treatment of trauma-related disorders. *Frontiers in psychiatry*, *13*, 863552.
Oehen, P., Traber, R., Widmer, V., & Schnyder, U. (2013). A randomized, controlled pilot study of MDMA (±3, 4-Methylenedioxymethamphetamine)-assisted psychotherapy for treatment of resistant, chronic Post-Traumatic Stress Disorder (PTSD). *Journal of psychopharmacology*, *27*(1), 40-52.
Olson, J. A., Suissa-Rocheleau, L., Lifshitz, M., Raz, A., & Veissiere, S. P. L. (2020). Tripping on nothing: placebo psychedelics and contextual factors. *Psychopharmacology (Berl)*, *237*(5), 1371-1382. https://doi.org/10.1007/s00213-020-05464-5
Palmer, M., & Maynard, O. M. (2022). Are you tripping comfortably? Investigating the relationship between harm reduction and the psychedelic experience. *Harm reduction journal*, *19*(1), 81.
Perkins, D., Schubert, V., Simonova, H., Tofoli, L. F., Bouso, J. C., Horak, M., Galvao-Coelho, N. L., & Sarris, J. (2021). Influence of Context and Setting on the Mental Health and Wellbeing Outcomes of Ayahuasca Drinkers: Results of a Large International Survey. *Front Pharmacol*, *12*, 623979. https://doi.org/10.3389/fphar.2021.623979
Phelps, J., & Henry, J. (2021). Foundations for training psychedelic therapists. In *Disruptive Psychopharmacology* (pp. 93-109). Springer.
Pontual, A. A. D., Tofoli, L. F., Collares, C. F., Ramaekers, J. G., & Corradi-Webster, C. M. (2021). The Setting Questionnaire for the Ayahuasca Experience: Questionnaire Development and Internal Structure. *Front Psychol*, *12*, 679016. https://doi.org/10.3389/fpsyg.2021.679016
Posternak, M. A., & Zimmerman, M. (2007). Therapeutic effect of follow-up assessments on antidepressant and placebo response rates in antidepressant efficacy trials. *British Journal of Psychiatry*, *190*(4), 287-292. https://doi.org/10.1192/bjp.bp.106.028555
Preller, K. H., Herdener, M., Pokorny, T., Planzer, A., Kraehenmann, R., Stämpfli, P., Liechti, M. E., Seifritz, E., & Vollenweider, F. X. (2017). The fabric of meaning and subjective effects in LSD-induced states depend on serotonin 2A receptor activation. *Current Biology*, *27*(3), 451-457.
Priebe, S., Conneely, M., McCabe, R., & Bird, V. (2019). What can clinicians do to improve outcomes across psychiatric treatments: a conceptual review of non-specific components. *Epidemiol Psychiatr Sci*, *29*, e48. https://doi.org/10.1017/S2045796019000428

Rea, K., & Wallace, B. (2021). Enhancing equity-oriented care in psychedelic medicine: Utilizing the EQUIP framework. *International Journal of Drug Policy*, *98*, 103429.
Robinson, L., Delgadillo, J., & Kellett, S. (2020). The dose-response effect in routinely delivered psychological therapies: A systematic review. *Psychotherapy research*, *30*(1), 79-96.
Romeo, B., Kervadec, E., Fauvel, B., Strika-Bruneau, L., Amirouche, A., Verroust, V., Piolino, P., & Benyamina, A. (2024). Safety and risk assessment of psychedelic psychotherapy: a meta-analysis and systematic review. *Psychiatry Research*, 115880.
Roseman, L., Erritzoe, D., Nutt, D., Carhart-Harris, R., & Timmermann, C. (2024). Interrupting the Psychedelic Experience Through Contextual Manipulation to Study Experience Efficacy. *JAMA Netw Open*, *7*(7), e2422181. https://doi.org/10.1001/jamanetworkopen.2024.22181
Rosenblat, J. D., Meshkat, S., Doyle, Z., Kaczmarek, E., Brudner, R. M., Kratiuk, K., Mansur, R. B., Schulz-Quach, C., Sethi, R., Abate, A., Ali, S., Bawks, J., Blainey, M. G., Brietzke, E., Cronin, V., Danilewitz, J., Dhawan, S., Di Fonzo, A., Di Fonzo, M., . . . McIntyre, R. S. (2024). Psilocybin-assisted psychotherapy for treatment resistant depression: A randomized clinical trial evaluating repeated doses of psilocybin. *Med*, *5*(3), 190-200 e195. https://doi.org/10.1016/j.medj.2024.01.005
Ryherd, E. E., West, J. E., Busch-Vishniac, I. J., & Waye, K. P. (2008). Evaluating the hospital soundscape. *Acoustics today*, *4*(4), 22-29.
Sabina, M. (2003). *María Sabina: Selections* (Vol. 2). Univ of California Press.
Sapoznikow, A., Walsh, Z., Tupper, K. W., Erowid, E., & Erowid, F. (2019). The influence of context on Ayahuasca experiences: an analysis of experience reports. *Journal of Psychedelic Studies*, *3*(3), 288-294.
Short, A. E., Short, K. T., Holdgate, A., Ahern, N., & Morris, J. (2011). Noise levels in an Australian emergency department. *Australasian Emergency Nursing Journal*, *14*(1), 26-31.
Skiles, Z., Dixon, J. R., Friedrich, D., Reed, D., & Stauffer, C. S. (2023). Peer support and psychedelics. *Journal of Military, Veteran and Family Health*, *9*(5), 80-87.
Sloshower, J., Guss, J., Krause, R., Wallace, R. M., Williams, M. T., Reed, S., & Skinta, M. D. (2020). Psilocybin-assisted therapy of major depressive disorder using acceptance and commitment therapy as a therapeutic frame. *Journal of Contextual Behavioral Science*, *15*, 12-19.
Stein, D. M., & Lambert, M. J. (1984). On the relationship between therapist experience and psychotherapy outcome. *Clinical Psychology Review*, *4*(2), 127-142.
Strassman, R. J. (1984). Adverse reactions to psychedelic drugs. A review of the literature. *The Journal of nervous and mental disease*, *172*(10), 577-595.
Studerus, E., Gamma, A., Kometer, M., & Vollenweider, F. X. (2012). Prediction of psilocybin response in healthy volunteers. *PLoS One*, *7*(2), e30800. https://doi.org/10.1371/journal.pone.0030800
Swanson, L. R. (2018). Unifying theories of psychedelic drug effects. *Frontiers in Pharmacology*, 172.
Taylor, K. (2017). The ethics of caring: Finding right relationship with clients. *Hanford Mead*.
Thal, S. B., Wieberneit, M., Sharbanee, J. M., Skeffington, P. M., Baker, P., Bruno, R., Wenge, T., & Bright, S. J. (2022). Therapeutic (Sub) stance: Current practice and therapeutic conduct in preparatory sessions in substance-assisted psychotherapy—A systematized review. *Journal of psychopharmacology*, *36*(11), 1191-1207.
Vogt, S. B., Ley, L., Erne, L., Straumann, I., Becker, A. M., Klaiber, A., Holze, F., Vandersmissen, A., Mueller, L., & Duthaler, U. (2023). Acute effects of intravenous DMT in a randomized placebo-controlled study in healthy participants. *Translational Psychiatry*, *13*(1), 172.
Wampold, B. E., & Imel, Z. E. (2015). *The great psychotherapy debate: The evidence for what makes psychotherapy work*. Routledge.
Watts, R. (2021). Psilocybin for depression: The ACE model manual.
Watts, R., & Luoma, J. B. (2020). The use of the psychological flexibility model to support psychedelic assisted therapy. *Journal of Contextual Behavioral Science*, *15*, 92-102.
Williams, K., & Brant, S. (2023). Tending a vibrant world: Gift logic and sacred plant medicines. *History of Pharmacy and Pharmaceuticals*, *65*(1), 8-32.
Williams, M. T., & Labate, B. C. (2020). Diversity, equity, and access in psychedelic medicine. *Journal of Psychedelic Studies*, *4*(1), 1-3.
Williams, M. T., Reed, S., & Aggarwal, R. (2020). Culturally informed research design issues in a study for MDMA-assisted psychotherapy for posttraumatic stress disorder. *Journal of Psychedelic Studies*, *4*(1), 40-50.
Wsół, A. (2023). Cardiovascular safety of psychedelic medicine: current status and future directions. *Pharmacological Reports*, *75*(6), 1362-1380.
Zeifman, R. J., Kettner, H., Ross, S., Weiss, B., Mithoefer, M. C., Mithoefer, A. T., & Wagner, A. C. (2024). Preliminary evidence for the importance of therapeutic alliance in MDMA-assisted psychotherapy for posttraumatic stress disorder. *European Journal of Psychotraumatology*, *15*(1), 2297536.

Mithoefer M (2017). A manual for MDMA-assisted psychotherapy in the treatment of posttraumatic stress disorder; Version 8. http://www.maps.org/research/mdma/mdma-research-timeline/4887-a-manual-for-mdma-assisted-psychotherapy-in-the-treatment-of-ptsd.

MAPS (2019). Public Announcement of Ethical Violation by Former MAPS-Sponsored Investigators. https://maps.org/2019/05/24/statement-public-announcement-of-ethical-violation-by-former-maps-sponsored-investigators/.
