# Supplementary material for: An international Delphi consensus for reporting of setting in psychedelic clinical trials
Source: Nat Med. 2025 Jun 3;31(7):2186–95. doi: 10.1038/s41591-025-03685-9 (PMC12283393; doi:10.1038/s41591-025-03685-9)
Supplement: Supplementary file 2 — Reporting Summary [file 41591_2025_3685_MOESM2_ESM.pdf]

nature portfolio

Corresponding author(s): Chloé Pronovost-Morgan

Last updated by author(s): Mar 24, 2025

Reporting Summary

Nature Portfolio wishes to improve the reproducibility of the work that we publish. This form provides structure for consistency and transparency in reporting. For further information on Nature Portfolio policies, see our Editorial Policies and the Editorial Policy Checklist.

Statistics

For all statistical analyses, confirm that the following items are present in the figure legend, table legend, main text, or Methods section.

n/a	Confirmed	
☐	☒	The exact sample size (n) for each experimental group/condition, given as a discrete number and unit of measurement
☐	☒	A statement on whether measurements were taken from distinct samples or whether the same sample was measured repeatedly
☒	☐	The statistical test(s) used AND whether they are one- or two-sided<br>*Only common tests should be described solely by name; describe more complex techniques in the Methods section.*
☒	☐	A description of all covariates tested
☒	☐	A description of any assumptions or corrections, such as tests of normality and adjustment for multiple comparisons
☐	☒	A full description of the statistical parameters including central tendency (e.g. means) or other basic estimates (e.g. regression coefficient) AND variation (e.g. standard deviation) or associated estimates of uncertainty (e.g. confidence intervals)
☒	☐	For null hypothesis testing, the test statistic (e.g. F, t, r) with confidence intervals, effect sizes, degrees of freedom and P value noted<br>*Give P values as exact values whenever suitable.*
☒	☐	For Bayesian analysis, information on the choice of priors and Markov chain Monte Carlo settings
☒	☐	For hierarchical and complex designs, identification of the appropriate level for tests and full reporting of outcomes
☒	☐	Estimates of effect sizes (e.g. Cohen's d, Pearson's r), indicating how they were calculated

Our web collection on statistics for biologists contains articles on many of the points above.

Software and code

Policy information about availability of computer code

Data collection	Qualtrics-based surveys were used for data collection.
Data analysis	Data was analyzed in Microsoft Excel and plots were generated in Excel and R (RStudio Version 2021-09-0+351).

For manuscripts utilizing custom algorithms or software that are central to the research but not yet described in published literature, software must be made available to editors and reviewers. We strongly encourage code deposition in a community repository (e.g. GitHub). See the Nature Portfolio guidelines for submitting code & software for further information.

Data

Policy information about availability of data

All manuscripts must include a data availability statement. This statement should provide the following information, where applicable:

- Accession codes, unique identifiers, or web links for publicly available datasets
- A description of any restrictions on data availability
- For clinical datasets or third party data, please ensure that the statement adheres to our policy

Given the partially anonymous nature of Delphi studies and the personal opinions shared by participants, data will not be deposited in a public repository. Rather, deidentified individual-level data, along with additional study materials such as study surveys, will be made available upon reasonable request for a minimum of five years following publication. Requests should be directed to the corresponding author Chloé Pronovost-Morgan, at chloe.pronovost-morgan@mail.mcgill.ca, and include the following information: a brief proposal including study aims, data requested, planned analysis, and credentials of the requestor(s). All requests will be

reviewed by the study lead authors based on scientific merit, feasibility, and ethical considerations. An initial response will be provided within two weeks. For accepted proposals, data sharing will be contingent on an executed data transfer agreement.

Research involving human participants, their data, or biological material

Policy information about studies with human participants or human data. See also policy information about sex, gender (identity/presentation), and sexual orientation and race, ethnicity and racism.

Reporting on sex and gender	Study participants were asked to report the gender they identify with (the options given were: “male”, “female”, “non-binary”, “prefer to self-describe” with a free-text box to specify, or “prefer not to say”). Participants were not asked about their biological sex. This data was collected to assess the diversity of the study participants and to explore whether perspectives differed across different groups.
Reporting on race, ethnicity, or other socially relevant groupings	Study participants were first asked if they identify as Indigenous, with the response options “Yes”, “No” and “prefer not to say”. If they responded “Yes”, they could specify the name of their Nation/tribe/peoples in a free-text box. Participant were then asked what best describes their ethnic origin, and were provided with the following classification terms: “White”; “Black / African /Caribbean”; “Asian (Indian, Pakistani, Bangladeshi, Chinese, any other Asian background)”; “Hispanic / Latino”; “Middle Eastern or North African”; “Mixed two or more ethnic groups”; “Prefer not to say”, as well as “Not listed above, I identify as” with a free-text box to specify. This data was also collected to assess the diversity of the study participants and to explore whether perspectives differed across different groups.
Population characteristics	Beyond their gender identify and ethnic origins, study participants were asked the following demographic information: age (categorical variable in increments of 5 from 18 to 65+ years old); country of origin (drop down menu with all countries to choose from); native language; current marital status (married, living with a partner, widowed, divorced/separated, never married); religion (options: Christian, Muslim, Buddhist, Jewish, Hindu, Atheist or agnostic, Spiritual but not religious, Prefer not to say, Not listed above, I identify as (with a free-text box); highest level of education (ranging from “none” to “doctorate or professional degree”; and employment status. For each question, participants had the option to choose “prefer not to say”. This data was collected to generate a portrait of the demographic characteristics of the study participants. Participants were also asked to describe their professional affiliations and areas of expertise. These demographic variables were collected to understand the composition of the study sample. They also served as source for secondary analyses; namely, a regional sub-group analysis conducted during the peer review process.
Recruitment	Clinicians and researchers were identified through searches of the academic literature, conference websites, psychedelic-related institutes or organizations, and the authors’ academic networks. Snowball sampling was also used after initial invitations were sent. Expertise was validated based on academic and professional qualifications, years of relevant experience, and contributions to the field. Past trial participants were primarily recruited in collaboration with the co-founders of the Psychedelic Participant Advocacy Network (PsyPAN), a non-profit organization based in the UK. The PsyPAN co-founders informed members about the study, and those who showed interest were put into contact with the study leads. Efforts were made to recruit a diverse and representative sample by targeting a broad range of professionals across institutions, regions, and disciplines, and by leveraging networks that include individuals with varied backgrounds, including “expertise by experience”.
Ethics oversight	This study was conducted in accordance with the ethical guidelines set forth by the Imperial College Research Ethics Committee, which granted the study approval (reference number 6568054). All study participants provided online written consent at the beginning of the first survey. During data collection, participants were quasi-anonymous, i.e., anonymous to other experts but identifiable to the study team. After data collection, personal identifiers were replaced with unique participant identification numbers, with the identifying key encrypted and stored separately.

Note that full information on the approval of the study protocol must also be provided in the manuscript.

Field-specific reporting

Please select the one below that is the best fit for your research. If you are not sure, read the appropriate sections before making your selection.

☐ Life sciences ☒ Behavioural & social sciences ☐ Ecological, evolutionary & environmental sciences

For a reference copy of the document with all sections, see nature.com/documents/nr-reporting-summary-flat.pdf

Behavioural & social sciences study design

All studies must disclose on these points even when the disclosure is negative.

Study description	This study employed the Delphi method, an iterative, consensus-based survey technique, to develop the Reporting of Setting in Psychedelic Clinical Trials (ReSPCT) guidelines. The data generated are mixed-methods.
Research sample	The sample consisted of 89 experts from 17 countries and 50 institutions, including clinicians, researchers, and past psychedelic clinical trial participants who were chosen for their credentials and expertise in in at least one of the following related areas: psychopharmacology, neuroimaging, psychedelic-assisted therapy, clinical trial design, and/or harm reduction. The majority identified as male (53.9%), followed by female (40.4%) and non-binary (4.5%). Most participants were between 35-44 years old (37.1%) or 45-54 years old (23.6%). Ethnically, most identified as White (74.2%), with smaller representations from Indigenous (7.9%), Asian (3.4%), Middle Eastern or North African (3.4%), and other groups. The majority held doctorate or professional degrees

(67.0%). The study's focus on clinical trials and regulated psychedelic research meant that perspectives from different settings were less represented. The sample primarily consisted of mid-career professionals from Western countries, where psychedelic research is most active. As a result, the sample reflected existing demographic trends in the field, including a lack of ethnic/racial diversity among researchers and trial participants; a limitations we acknowledge in the manuscript.

Sampling strategy	Sample size calculations are not typically done in Delphi studies, as the goal is not to generalize findings to a broader population but to reach agreement among experts. Although there is no standard sample size for Delphi studies (Diamond et al., 2014), most include up to 25 participants, and guidelines recommend at least 15 to 20 experts in any given subgroup for statistical purposes (Beiderbeck et al., 2021). Accordingly, we aimed to recruit groups of 20 researchers, clinicians, and past trial participants, for a total of 60 experts by Round Four. Taking into account attrition, we sought enrolment of 90 experts for Round One.
Data collection	Data were collected through four iterative online survey rounds on the platform Qualtrics. Participants rated the importance of proposed reporting items and provided qualitative feedback to refine the guidelines. Consensus was determined based on predefined statistical thresholds (70% of experts or more rating an item as "important" or "very important" on a 7-point Likert scale). Participants completed surveys independently, and no one besides the three study leads had access to the responses. Given the online, survey-based nature of data collection, there was no in-person researcher-participant interaction, and no observers were present while participants entered their responses to the surveys. The study leads were not blinded to the study hypothesis, as Delphi studies rely on the study leads to summarize and feedback expert-driven data over the course of multiple study rounds with the aim of reaching an expert-based consensus. However, participants were blind to other respondents' identities to minimize influence throughout the study.
Timing	The study was conducted between April and August 2023. Each round was approximately 1 month long, with 1-2 weeks reserved for intermediate analyses.
Data exclusions	Potential participants were excluded if they were under 18 or did not meet the predefined expertise criteria. 'Experts' were defined as individuals with specialized knowledge or skills derived from training or lived experience. More specifically, participants required relevant academic or professional qualifications and to have made meaningful contributions to the field (e.g., publications, presentations, or equivalent). Eligible experts also had to demonstrate knowledge or experience in at least one of the following areas: psychopharmacology, neuroimaging, psychedelic-assisted therapy, clinical trial design, or harm reduction. Those who did not meet these criteria were excluded. Those who did were invited to take part in the study.
Non-participation	Personalized email invitations to participate were initially sent to 149 experts, followed by an additional 34 experts identified by snowball recruitment, for a total of N = 183 invited experts. Among them, n = 85 did not respond to the invitation or follow-up email, and thus were deemed not interested in taking part, and n = 9 refused the invitation (5 for time constraints, 1 for disagreeing with the chosen study method [Delphi] and 4 for personal/undisclosed reasons). A total of 89 experts (48·6%) consented to participate and all fully completed the first round. Thereafter, 73 experts completed Round 2, 68 experts completed Round 3, and 62 experts completed Round 4, yielding a 30% attrition rate between the first and final round.
Randomization	Randomization was not applicable as Delphi studies rely on expert consensus rather than comparing experimental conditions. The aim of the Delphi method is to generate a consensus among pre-selected experts, rather than testing the efficacy of an intervention in one group compared to another.

Reporting for specific materials, systems and methods

We require information from authors about some types of materials, experimental systems and methods used in many studies. Here, indicate whether each material, system or method listed is relevant to your study. If you are not sure if a list item applies to your research, read the appropriate section before selecting a response.

Materials & experimental systems

n/a	Involved in the study
☒	☐ Antibodies
☒	☐ Eukaryotic cell lines
☒	☐ Palaeontology and archaeology
☒	☐ Animals and other organisms
☒	☐ Clinical data
☒	☐ Dual use research of concern
☒	☐ Plants

Methods

n/a	Involved in the study
☒	☐ ChIP-seq
☒	☐ Flow cytometry
☒	☐ MRI-based neuroimaging

Plants

Seed stocks	*Report on the source of all seed stocks or other plant material used. If applicable, state the seed stock centre and catalogue number. If plant specimens were collected from the field, describe the collection location, date and sampling procedures.*
Novel plant genotypes	*Describe the methods by which all novel plant genotypes were produced. This includes those generated by transgenic approaches, gene editing, chemical/radiation-based mutagenesis and hybridization. For transgenic lines, describe the transformation method, the number of independent lines analyzed and the generation upon which experiments were performed. For gene-edited lines, describe the editor used, the endogenous sequence targeted for editing, the targeting guide RNA sequence (if applicable) and how the editor was applied.*
Authentication	*Describe any authentication procedures for each seed stock used or novel genotype generated. Describe any experiments used to assess the effect of a mutation and, where applicable, how potential secondary effects (e.g. second site T-DNA insertions, mosiacism, off-target gene editing) were examined.*
